# Mammalian Rest/Activity Patterns Explained by Physiologically Based Modeling

**DOI:** 10.1371/journal.pcbi.1003213

**Published:** 2013-09-05

**Authors:** A. J. K. Phillips, B. D. Fulcher, P. A. Robinson, E. B. Klerman

**Affiliations:** 1 Division of Sleep Medicine, Brigham & Women's Hospital, Harvard Medical School, Boston, Massachusetts, United States of America; 2 Department of Physics, Oxford University, Clarendon Laboratory, Oxford, Oxfordshire, United Kingdom; 3 School of Physics, University of Sydney, Sydney, New South Wales, Australia; 4 Brain Dynamics Center, Sydney Medical School - Western, University of Sydney, Westmead, New South Wales, Australia; 5 Center for Integrated Research and Understanding of Sleep, Sydney, New South Wales, Australia; University of Notre Dame, United States of America

## Abstract

Circadian rhythms are fundamental to life. In mammals, these rhythms are generated by pacemaker neurons in the suprachiasmatic nucleus (SCN) of the hypothalamus. The SCN is remarkably consistent in structure and function between species, yet mammalian rest/activity patterns are extremely diverse, including diurnal, nocturnal, and crepuscular behaviors. Two mechanisms have been proposed to account for this diversity: (i) modulation of SCN output by downstream nuclei, and (ii) direct effects of light on activity. These two mechanisms are difficult to disentangle experimentally and their respective roles remain unknown. To address this, we developed a computational model to simulate the two mechanisms and their influence on temporal niche. In our model, SCN output is relayed via the subparaventricular zone (SPZ) to the dorsomedial hypothalamus (DMH), and thence to ventrolateral preoptic nuclei (VLPO) and lateral hypothalamus (LHA). Using this model, we generated rich phenotypes that closely resemble experimental data. Modulation of SCN output at the SPZ was found to generate a full spectrum of diurnal-to-nocturnal phenotypes. Intriguingly, we also uncovered a novel mechanism for crepuscular behavior: if DMH/VLPO and DMH/LHA projections act cooperatively, daily activity is unimodal, but if they act competitively, activity can become bimodal. In addition, we successfully reproduced diurnal/nocturnal switching in the rodent *Octodon degu* using coordinated inversions in both masking and circadian modulation. Finally, the model correctly predicted the SCN lesion phenotype in squirrel monkeys: loss of circadian rhythmicity and emergence of ∼4-h sleep/wake cycles. In capturing these diverse phenotypes, the model provides a powerful new framework for understanding rest/activity patterns and relating them to underlying physiology. Given the ubiquitous effects of temporal organization on all aspects of animal behavior and physiology, this study sheds light on the physiological changes required to orchestrate adaptation to various temporal niches.

## Author Summary

Controlled timing of all daily activity patterns is a highly adaptive trait that allows an animal to exploit its particular ecological environment. Environmental pressures such as light cycles, temperature cycles, food availability, and timing of predator activity selectively shape the activity patterns of different species. Mammalian species are remarkably diverse and flexible in their daily activity patterns, including a spectrum of diurnal (day-active) to nocturnal (night-active) and crepuscular (dawn/dusk-active) patterns. Despite the importance of activity patterns to all aspects of behavior and physiology, the mechanisms that underlie these patterns are not well understood. Using a computational model, we demonstrated that a wide array of activity phenotypes can be captured in terms of just two hypothesized mechanisms: reshaping of the output of the brain's master circadian clock, and direct responses to light exposure. This work links together a wide range of mammalian behaviors and sheds new light on their potential evolutionary underpinnings.

## Introduction

Sleep recordings in 127 mammalian species [1] have revealed a rich array of phenotypes with respect to the temporal organization of rest/activity and sleep/wake patterns [2]. These phenotypic differences between species are thought to reflect evolutionary adaptations to specific temporal niches (the times at which an animal is usually active) [3]. In mammals, temporal niches span a continuum from diurnal (day-active) to cathemeral (no time-of-day preference) to nocturnal (night-active) [4]. In addition, the shape of the activity waveform varies; some species are active in a single daily peak, while others have two or more daily peaks, e.g., crepuscular (dawn- and dusk-active) animals. Temporal niche can also change seasonally, developmentally, or in response to other environmental stimuli [5], [6], [7]. Remarkably, some rodents switch from diurnal to nocturnal in the laboratory when provided access to a running wheel [8], [9], [10]. Our goal is to develop a theoretical framework to compare these diverse phenotypes and quantitatively relate them to a small number of underlying physiological mechanisms.

**Figure 1:**
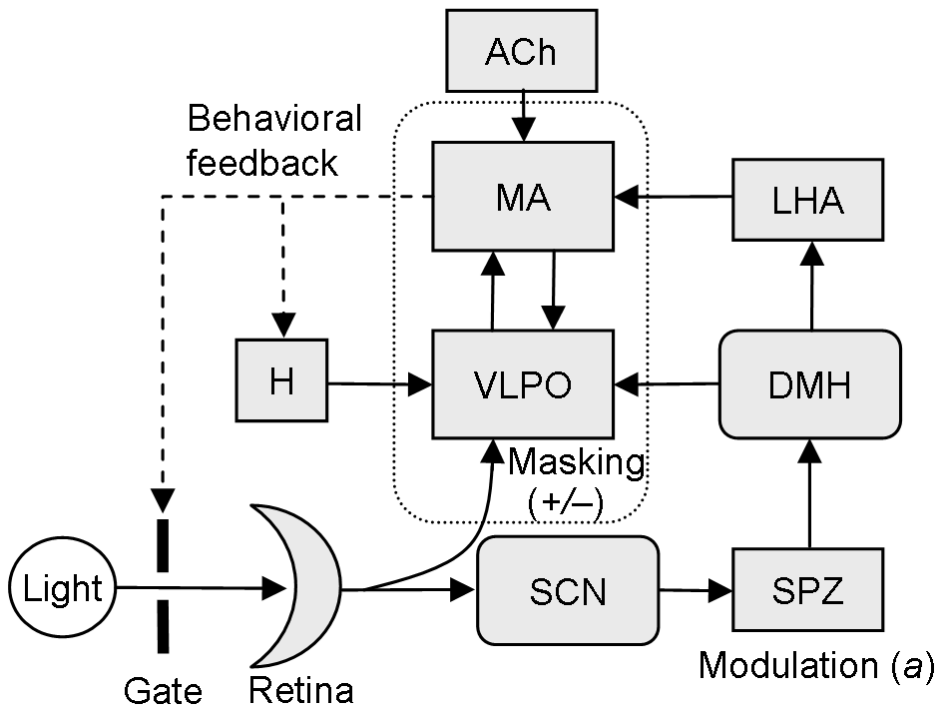
Schematic of our physiologically based computational model. Retinal light input is projected to the suprachiasmatic nucleus (SCN) and ventrolateral preoptic area (VLPO). Mutual inhibition between the sleep-promoting VLPO and wake-promoting monoaminergic (MA) nuclei forms the basis of the sleep/wake switch (outlined). During wake, MA firing rates are high and VLPO firing rates are low. SCN output is relayed to the sleep/wake switch via the subparaventricular zone (SPZ), dorsomedial hypothalamus (DMH), and orexinergic neurons in the lateral hypothalamus (LHA). The MA also receives excitatory cholinergic input (ACh). A homeostatic sleep drive (H) to the VLPO is modeled. Wake/sleep state and rest/activity are defined by MA firing rate, 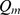, which provides behavioral feedback to H and gates environmental light.

Recently, physiological mechanisms that control the timing of rest/activity and sleep/wake patterns have been identified. Chief among these is the master circadian pacemaker in the suprachiasmatic nucleus (SCN) of the hypothalamus [11]. Surprisingly, however, there are no systematic differences in circadian function between nocturnal and diurnal mammals [12]. In both groups, the SCN is similar in structure [13] and has highest neuronal firing rates during circadian daytime [14]. Furthermore, melatonin secretion, which is under SCN control in mammals, always occurs during circadian nighttime [15]. Therefore, temporal niche must be largely determined by mechanisms outside of the SCN, including downstream *modulation* of SCN output [16]. As shown in **Figure 1**, the SCN projects to the subparaventricular zone (SPZ), with subsequent relays to the dorsomedial hypothalamus (DMH), and from there to nuclei that regulate sleep/wake and rest/activity, including the ventrolateral preoptic area (VLPO) and orexinergic neurons of the lateral hypothalamic area (LHA) [11]. Experimental results confirm that SCN output is inverted in nocturnal animals at the SPZ [17]. This results in inversions in neuronal activity patterns in many downstream wake-promoting regions [18] and physiologic outputs [19], relative to diurnal animals.

The acute effect of light on behavior, i.e., *masking* of circadian rhythms, also shapes the temporal organization of rest/activity patterns [20]. Typically, light exposure increases vigilance in diurnal animals (positive masking) and decreases vigilance in nocturnal animals (negative masking). Masking strength depends on the measure of vigilance used, with stronger effects of light on waking behaviors such as locomotor activity and feeding than on time spent awake [21], [22], [23]. The dominant pathway for masking is hypothalamic, as lesions that sever the retinohypothalamic tract abolish masking [24]. The intergeniculate leaflet and visual cortex modify masking strength, but neither structure is required for masking [25], [26]. Candidate pathways for masking thus include retinal input to the VLPO [27], pretectum [28], and SCN [29], [30]. Following SCN lesions, masking persists in some reports [31], [32], [33] and is weakened or abolished in others [34], [35]. We speculate that this inconsistency could reflect incidental damage to fibers passing near the SCN, such as retinal fibers to the VLPO or pretectum.

Studies of mammals that change their temporal niche have discovered a potential interaction between masking and circadian modulation. When Nile grass rats switch from diurnal to nocturnal behavior, they also switch from positive to negative masking [36]. The same phenomenon is observed in the degu, a normally diurnal rodent [37]. However, degus can also express a stable intermediate phenotype in which masking is inverted but the circadian rhythm is not [37]. These findings, along with lesion studies [6], show that changes in circadian modulation and masking can occur independently.

Untangling the relative contributions of masking and circadian modulation to rest/activity patterns is a challenging experimental task. Therefore, we developed a new physiologically based mathematical model that includes both mechanisms. Using this model, we were able to capture a wide array of experimentally-observed rest/activity patterns and relate them to underlying physiology. As shown in **Figure 1**, the model encompasses key sleep-regulatory nuclei in the brainstem and hypothalamus, including the mutually inhibitory sleep-promoting VLPO and wake-promoting monoaminergic (MA) nuclei, which together comprise the sleep/wake switch [38]. The DMH relays the circadian signal via two pathways: (i) to VLPO (the *DMH/VLPO relay*), and (ii) to LHA (the *DMH/LHA relay*). In the model, SCN output is modulated at the SPZ by a multiplicative factor, 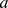, with 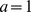 corresponding to diurnal (SCN and SPZ firing in phase) and 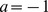 corresponding to nocturnal (circadian inversion, with SCN and SPZ firing out of phase). Masking is modeled as a direct retinal input to the VLPO, with an excitatory input corresponding to negative masking and an inhibitory input corresponding to positive masking. In this model, MA firing rate, 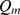, is used to distinguish between sleep (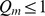 s^−1^) and wake (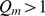 s^−1^). Higher MA firing rates are assumed to correspond to higher levels of activity, which is justified by experimental observations [39]. For more details of the model, see the **Methods** and **Text S1**.

## Results

### The Nocturnal-Diurnal Spectrum

The lack of correspondence between SCN firing patterns and diurnal/nocturnal preference indicates that other mechanisms must be involved in determining temporal niche. We hypothesized that modulation of SCN output by the SPZ could account for the degree to which an animal is either diurnal or nocturnal. We expected circadian modulation to be the dominant mechanism for this, rather than masking effects of light, because differences in the phasing of rest/activity patterns between diurnal and nocturnal species can persist even when they are free running in constant darkness. To examine the effects of SPZ modulation in isolation, we omitted masking, omitted the DMH/LHA relay, and simulated a rodent entrained to a 24-h light/dark (LD) cycle with 12 h of 100 lux (i.e., bright enough to achieve entrainment of the circadian rhythm to the LD cycle).

**Figure 2:**
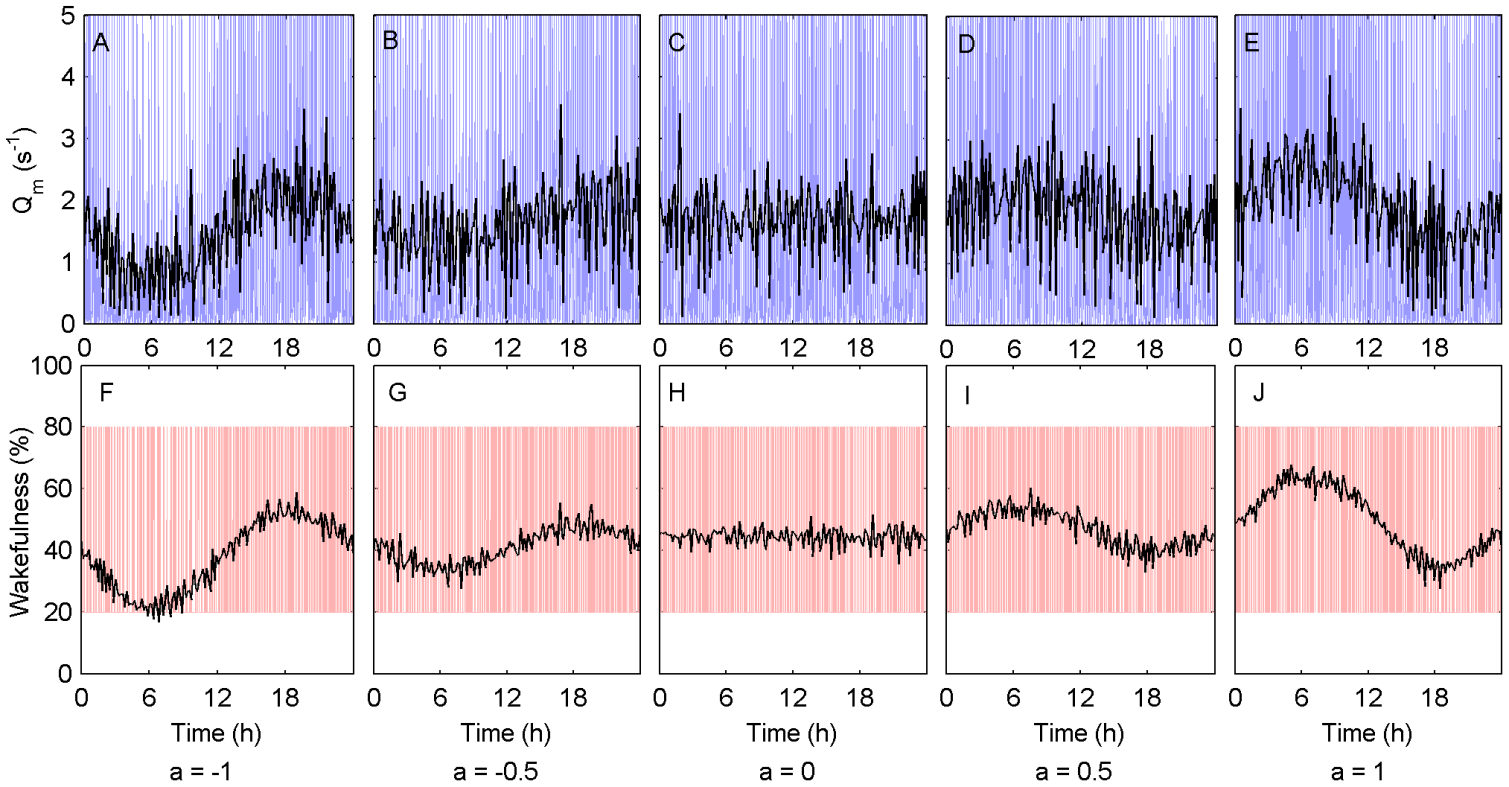
SPZ modulation of SCN output yields a spectrum of nocturnal to diurnal phenotypes. Model simulations of rodent sleep/wake patterns are shown for different values of the modulation parameter 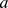, which takes values from −1 (nocturnal) to 1 (diurnal) going from left to right. The simulated rodent was under a 24-h light/dark cycle with 12 h of 100 lux followed by 12 h of 0 lux. Panels (A)–(E) show the MA firing rate, 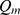, across a 24-h period (blue) and averaged across this period in 10-min non-overlapping windows (black). Wakefulness is defined as 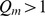 s^−1^. Panels (F)–(J) show arousal state across a 24-h period (red), with high values corresponding to wake and low values corresponding to sleep, as well as average percentage wakefulness (black), averaged across 30 days in 10-min non-overlapping windows.

With 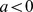, the SPZ fires out of phase with the SCN, resulting in a sleep-promoting circadian signal during the light phase. As shown in **Figure 2A** for 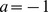, this results in higher MA firing rates (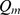) during the dark phase and a nocturnal phenotype. 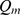 is not continuously high at any circadian phase, instead alternating frequently between high and low values across the day. As in previous work, the model is defined to be awake whenever 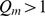 s^−1^. The rapidly alternating dynamics for 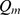 therefore correspond to polyphasic sleep/wake patterns, as typically seen in rodents. The time series for sleep/wake state is shown in **Figure 2F**, along with the average value (in 10 min windows, over 60 days), displaying a nocturnal phenotype. We note that the model includes noise to generate more realistic variability, but the polyphasic sleep/wake patterns in **Figure 2** and later sections are not dependent on the inclusion of noise; they are also a feature of the deterministic dynamics for these parameter values [40].

Increasing the value of 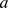 results in a continuous variation in phenotype from strongly nocturnal for 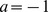 (**Figure 2A and 2F**) to slightly nocturnal for 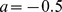 (**Figure 2B and 2G**) to cathemeral for 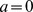 (**Figure 2C and 2H**) to slightly diurnal for 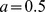 (**Figure 2D and 2I**) to strongly diurnal for 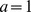 (**Figure 2E and 2J**). With 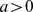, the SPZ fires in phase with the SCN, resulting in a wake-promoting circadian signal during the light phase. The average total wakefulness across the day also varies with 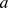, because SCN output does not promote wake and sleep symmetrically, as discussed below. Using our model, we thus showed that modulation of the circadian signal at the SPZ is a sufficient mechanism to explain much of the variability in temporal niche observed experimentally. However, this mechanism alone can not account for all differences in the daily activity waveform. The activity waveforms in **Figure 2** are all unimodal (i.e., have a single daily peak when time-averaged), whereas many species have two or more distinct activity peaks per day.

### Relay Interactions

**Figure 3:**
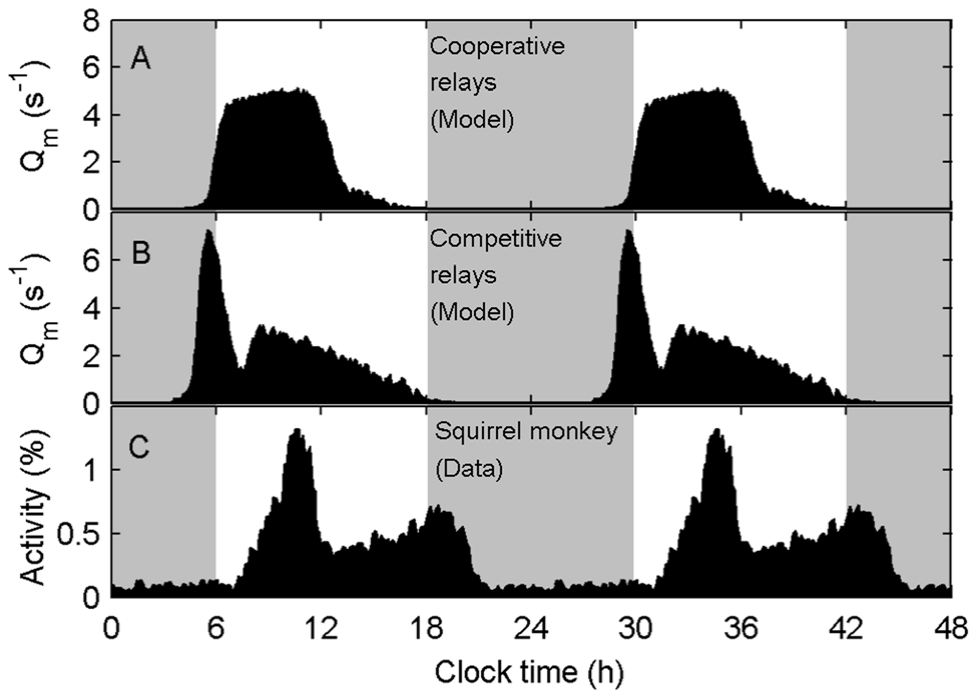
Cooperation and competition between DMH/VLPO and DMH/LHA relays result in unimodal and bimodal activity patterns, respectively. Simulated MA firing rate, 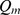, for a primate under a LD cycle, with 500 lux for 6–18 h and 0 lux for 18-6 h (shaded). The DMH/LHA pathway is (A) excitatory (i.e., cooperative), or (B) inhibitory (i.e., competitive). (C) The averaged activity patterns of a spider monkey living in the wild; the data are adapted from [12] and replotted manually here. Simulations and data are averaged in 5 min non-overlapping windows over 60 days and double-plotted.

Currently, it is unknown which physiological factors determine the shape of the daily activity waveform. We hypothesized that interactions between the two relay pathways, DMH/VLPO and DMH/LHA, could affect the shape of the waveform. In principle, the two pathways could be either cooperative or competitive in terms of their actions on the sleep/wake switch. Using our model, we simulated both types of interactions in a primate. We again omitted masking so as to examine this effect in isolation. When the pathways act cooperatively in relaying a diurnal signal (i.e., the DMH inhibits the sleep-promoting VLPO and excites the wake-promoting LHA), MA firing rates peak near the middle of the active phase (**Figure 3A**). However, when the pathways act competitively (i.e., the DMH inhibits both the VLPO and the LHA), MA firing rates have a bimodal waveform (**Figure 3B**). In the latter case, the dominant DMH/VLPO pathway maintains a diurnal sleep pattern, but orexinergic LHA neurons send a weak sleep-promoting signal near the middle of the wake episode. This results in a dip in activity early in the day, the timing of which is explained below. By varying the single model parameter corresponding to the synaptic connection strength from the DMH to LHA, we found that we are therefore able to change activity patterns from unimodal to bimodal. This is a heretofore unidentified mechanism for generating bimodal activity patterns. This finding justifies the apparent functional redundancy of multiple circadian relays; additional relays provide additional degrees of freedom for modulating the SCN output signal and behavior.

Bimodal waveforms are seen in many species; for reference we include the activity patterns of a spider monkey in **Figure 3C**. The timing and relative spacing of the peaks of activity are different between the simulation in **Figure 3B** and the experimental data in **Figure 3C**, with respect to the LD cycle. The data are shown only as an illustrative example of a mammalian species with a bimodal activity pattern; we do not attempt to achieve a best model fit. We have shown in previous work that the timing of the sleep/wake and rest/activity cycle can be modified with respect to the LD cycle by changing values of some of the parameters of the circadian and sleep homeostatic processes [41]. We do not pursue that idea further here.

**Figure 4:**
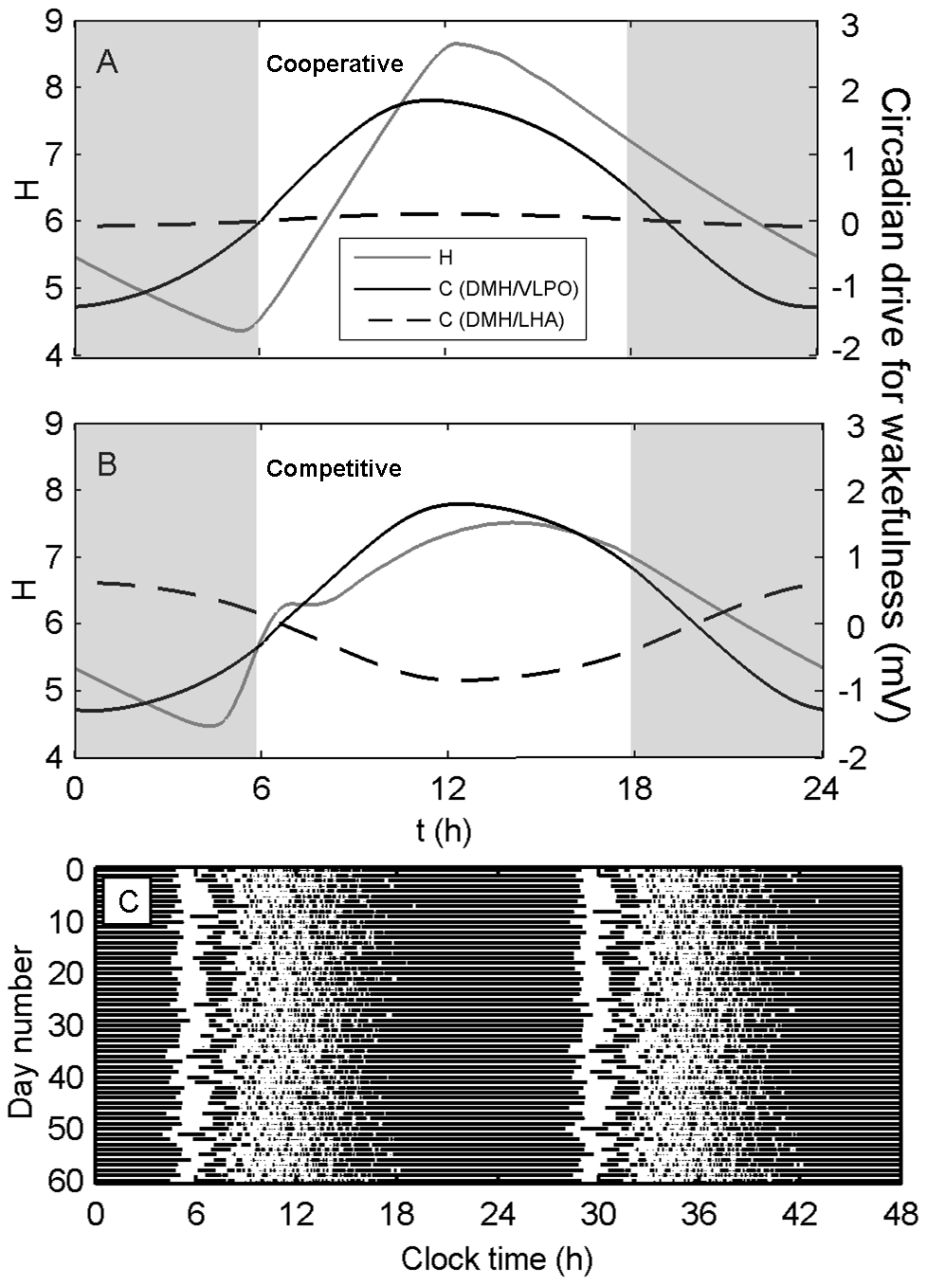
Circadian and homeostatic processes in cooperative versus competitive networks. The average waveforms (over 60 days) are shown for (A) cooperative and (B) competitive DMH/VLPO and DMH/LHA relays. Parameter values correspond to (A) and (B) in **Figure 3**, respectively. In each panel, we show the average waveform of the homeostatic drive for sleep (gray line), as well as the average waveforms of the circadian drives for wakefulness to both the VLPO via the DMH (black solid line) and the MA via the DMH/LHA (black dashed line). More positive values of the circadian drives for wakefulness correspond to greater inhibition of VLPO and greater excitation of MA for the DMH/VLPO and DMH/LHA relays, respectively. Panel (C) shows a double-plotted raster diagram of sleep (dark bars) over 60 days for the competitive case in panel (B).

The unimodal vs. bimodal dynamics can be better understood by examining the average waveforms of the circadian and sleep homeostatic processes in the cooperative and competitive cases. When the relays are cooperative (**Figure 4A**), both circadian drives for wakefulness – the DMH/VLPO and the DMH/LHA – peak near the middle of the light period, with the amplitude of the DMH/VLPO drive being much larger than the amplitude of the DMH/LHA drive. The homeostatic drive to sleep increases across the first half of the light period, when activity is high (**Figure 3A**), and decreases thereafter, when activity is low. When the relays are competitive (**Figure 4B**), the DMH/VLPO drive still promotes wakefulness during the light period and sleep during the dark period, but the DMH/LHA relay is now in anti-phase. This results in a weaker drive to sleep during the dark period, and therefore a slighter earlier awakening. Upon awakening, the net circadian drive is close to zero and the homeostatic drive for sleep is low, similar to the cooperative case. This results in an initial period of high activity (**Figure 3B**) and a rapid rise in sleep homeostatic pressure (**Figure 4B**). However, because the two circadian drives are in anti-phase, the overall circadian drive for wakefulness has lower amplitude and does not rise as quickly during the light period. Combined with the high homeostatic pressure, this results in a greater drive to sleep during the light period than in the cooperative case. As seen in **Figure 4C**, the combination of high homeostatic pressure and low circadian drive for wakefulness early in the light period results in a relatively consolidated block of sleep on most days. This nap is reflected in the flattening of the average homeostatic sleep drive early in the light period (**Figure 4B**); the reason the homeostatic pressure does not decrease more rapidly on average is because the timing of this nap is variable. In the deterministic case, shown in **Figure S1**, the timing of this nap is not variable, leading to a more consistent dip in average activity near the middle of the light period.

Following the early nap, homeostatic pressure is lower and the circadian drive for wakefulness is stronger. As shown in **Figure 4C**, this leads to an extended period of intermittent activity and wakefulness in the afternoon. This activity gradually transitions into nighttime sleep around a clocktime of 15 h, which corresponds to the turning point for the average homeostatic sleep drive. In many respects, this behavior is similar to that observed in spider monkeys [12], which show a robust peak in morning activity, followed by a period of low activity, which is then followed by a long afternoon period of intermittent activity. The main difference between those data and this model simulation is that the data show a slight increase in activity towards the end of the wake period, whereas our model predicts a gradually tailing off of activity across the afternoon for the parameter values used here.

### Temporal Niche Switching

**Figure 5:**
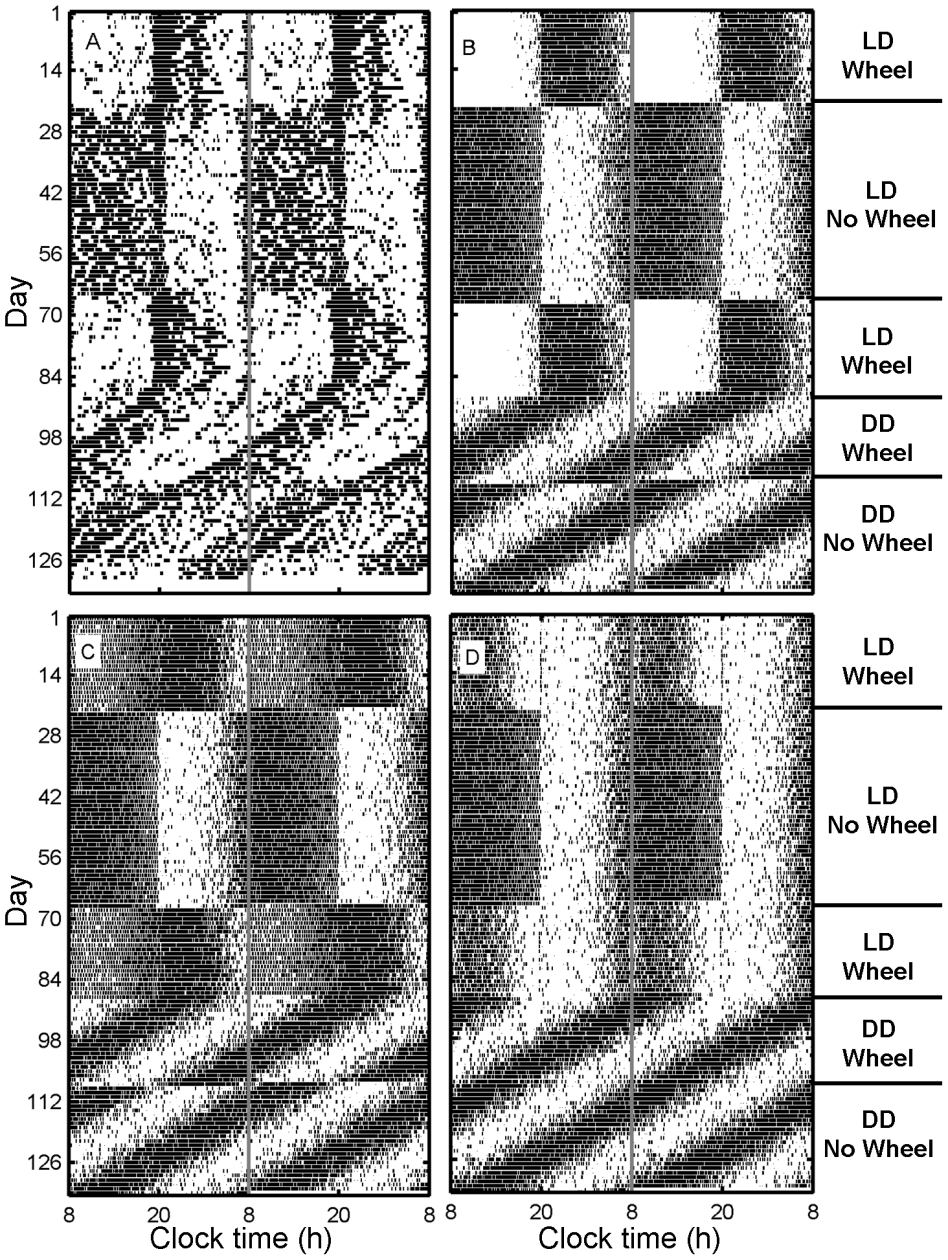
Experiments and simulations of temporal niche switching in degus. Temporal niche switching can be elicited in degus by the presence of a running wheel (days 1–22 and 68–108) in either LD (30 lux from clock time 8–20; days 1–88) or DD conditions (days 89-end). Panel (A) shows data from a single animal, with dark bars representing periods of above average core body temperature. Panels (B)–(D) show raster plots for single runs of the model using the same protocol, with dark bars representing periods of high activity (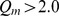 s^−1^), averaged in 10 min sliding windows. All raster plots are double-plotted. The effect of the running wheel is modeled by: (B) masking and circadian signal inversions, (C) circadian signal inversion, and (D) masking inversion. The data in panel (A) are adapted from [9] and manually replotted here.

While many species are solely nocturnal or solely diurnal, some species switch between these temporal niches under certain conditions. These switches are associated with inversions in circadian modulation and/or the masking response to light [37]. Currently, it is an open question whether temporal niche switching relies on simultaneous inversions in both of these factors, or whether inversions in circadian modulation alone are sufficient. We hypothesized that masking of activity by light must play a critical role in temporal niche switching. To test this hypothesis, we referred to the experiment of Kas and Edgar [9], where a diurnal degu inverted its activity patterns when provided access to a running wheel (**Figure 5A**). Switching was achieved almost immediately each time access to the running wheel was provided or removed. Switching was also achieved in constant darkness (DD), demonstrating that masking alone could not account for the phenomenon.

The model we developed provides the unique ability to infer the relative contributions of masking and circadian modulation from the observed behaviors. We simulated degu rest/activity patterns using model parameter values that correspond to a diurnal rodent. In the absence of a running wheel, we simulated a diurnal circadian signal (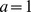) and positive masking of activity by light. To simulate the effects of introducing a running wheel, we modeled three possible physiological responses: (i) Wheel-induced inversion of both the circadian signal (from diurnal to nocturnal) and masking (from positive masking to negative masking), shown in **Figure 5B**; (ii) Wheel-induced inversion of just the circadian signal, shown in **Figure 5C**; (iii) Wheel-induced inversion of just masking, shown in **Figure 5D**. These responses to the running wheel were assumed to occur instantaneously. The two corresponding parameters (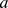 and the masking strength parameter) were the only parameters varied within the three simulations. Only three other model parameters were used to fit the model to this specific data set: intrinsic circadian period was set to 23.0 h to match the free-running period in DD; mean offset of the circadian signal was set to match daily activity duration under LD without a wheel; and circadian sensitivity to phase resetting by light was set sufficiently high to ensure entrainment to LD. All other model parameters had been constrained rigorously in previous work and took the values given in **Table S1**. As a model proxy for activity, we measured 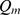 in sliding 10 min windows. Windows with 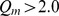 s^−1^ are plotted as bouts of high activity in **Figure 5**.

Simulating both masking inversion and circadian signal inversion (**Figure 5B**) yields a simulated activity record that is strikingly similar to the experimental data shown in **Figure 5A**. To our knowledge, this is the first reproduction of such a complex rest/activity phenotype by any mathematical model. Distinct transitions between diurnal and nocturnal phenotypes are reproduced under both LD and DD conditions. The model also reproduces multiple features to which it was not specifically fitted, including the extended duration of activity under LD without a wheel as compared to LD with a wheel, and the persistence of occasional activity bouts during the dark phase without the wheel and the light phase with the wheel. One notable discrepancy between these model simulations and data is the lack of a crepuscular activity profile under LD in the simulations; in the experimental data, activity peaks are seen at the times of lights on and off in LD, and at activity onset in DD.

The simulation using inversion of the circadian signal but not masking (**Figure 5C**) captures many features of the experimental data in **Figure 5A**, including inversion of activity in DD. A switch to primarily nocturnal activity under LD conditions in the presence of the wheel is also reproduced, but the transition is much less distinct than in the data. This is because masking remains positive, so the circadian signal and masking provide conflicting signals under LD conditions.

The simulation using inversion of masking but not the circadian signal (**Figure 5D**) fails to reproduce the inversion of activity patterns under DD conditions, because there is no light to respond to. Furthermore, under LD conditions, masking inversion is predicted to reduce activity during the light period, but not induce a complete inversion of rest/activity cycles. This is consistent with the results of a previous study in degus, in which a switch to negative masking (but no circadian inversion) under LD conditions resulted in no change to core body temperature during the dark phase, but significantly decreased temperature during the light phase [37]. In that instance, core body temperature during the light phase was similar to that of the fully nocturnal phenotype (i.e., both negative masking and circadian inversion); however, the light intensity used (350–400 lux) was considerably higher than in the Kas and Edgar study (30 lux). Our results therefore support the hypothesis that changes in masking and SCN output can occur independently, but they also demonstrate that, under these experimental conditions, inversions in the circadian signal and masking must occur together to achieve a complete temporal niche switch. These insights are gained by our novel strategy of reproducing rest/activity behavior from a mathematical model of the underlying physiology.

### SCN Lesions

Bilateral SCN lesions have been shown experimentally to result in fragmented and non-circadian rest/activity patterns [42]. Interestingly, these patterns are quasiperiodic, with a period of approximately 4 h in the squirrel monkey [43]. The reason for this periodicity is presently unknown. To investigate the cause of this phenotype, we simulated the experimental protocol of Edgar et al. [43], in which both intact and SCN-lesioned animals remained in 500 lux constant light (LL) conditions for several weeks. We simulated lesions by setting SCN output to zero (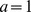 for intact, 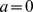 for SCN lesions). Positive masking by light was modeled in both cases, as this persists after SCN lesions in squirrel monkeys [31]. No other parameters were varied to simulate lesions. Only three other model parameters were used to fit the model to the data: the intrinsic circadian period was set to 25.2 h to match the LL data in the intact animal; the mean offset of the circadian signal was set to match daily wake duration in the intact animal; and the sleep homeostatic time constant was chosen to produce a primarily monophasic sleep pattern (i.e., one main sleep bout per day) in the intact animal, with similar consolidation to experimental data [44]. All other parameters took their nominal values, given in **Table S1**.

**Figure 6:**
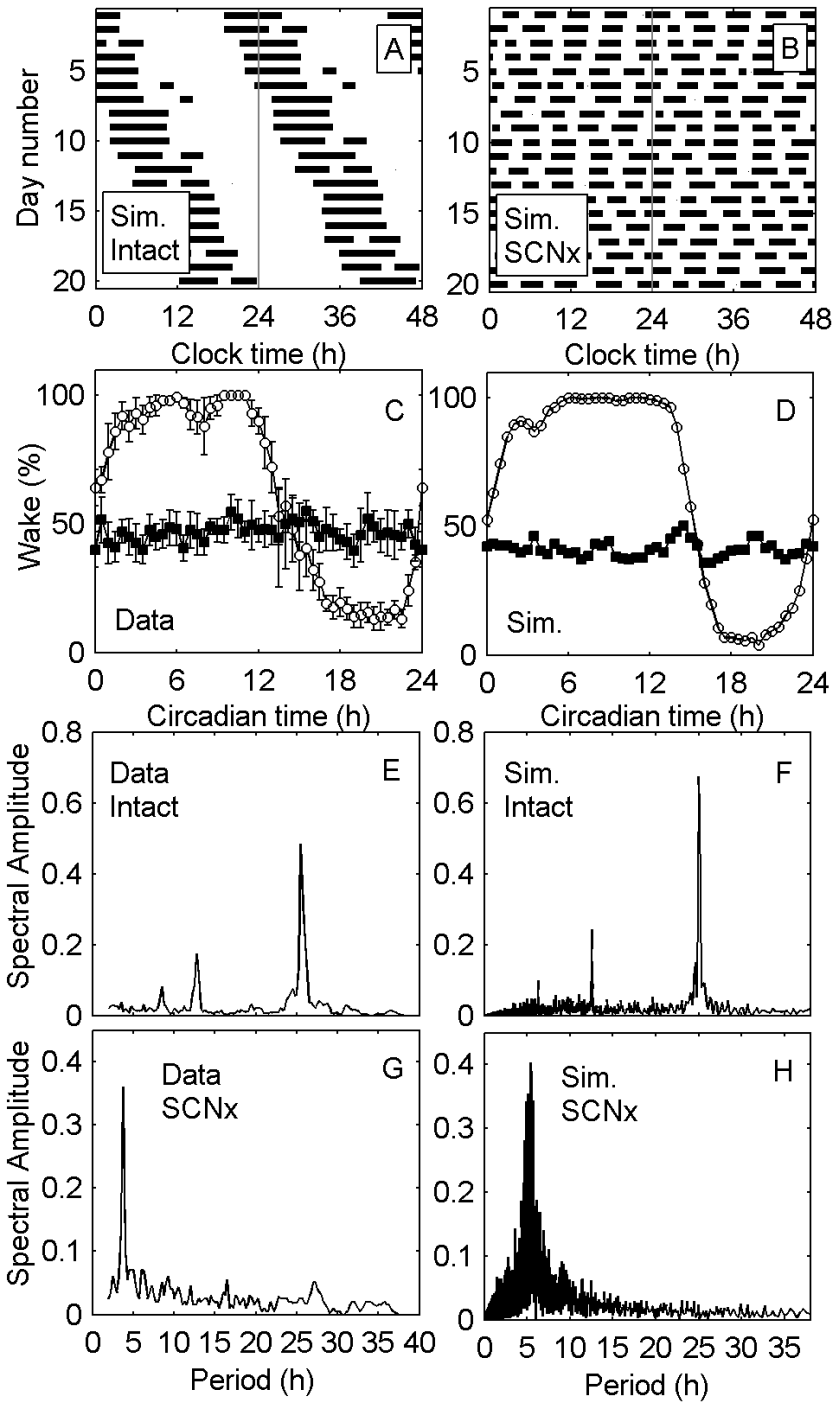
Simulations and data for SCN lesions in the squirrel monkey. Double-plotted raster plots of sleep/wake patterns are shown for (A) simulated intact animals and (B) simulated SCN-lesioned (SCNx) animals, with dark bars representing sleep (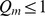 s^−1^). Percentage of time spent awake as a function of circadian time is shown for (C) data (mean ± SEM), and (D) simulation, for both intact (open circles) and lesioned (filled squares) animals. Circadian time is defined with respect to the 25.0-period of the intact animal, with 24 h of circadian time corresponding to one full circadian cycle. Circadian time zero is defined as the first bout with >50% average wake in the intact animal. Spectral amplitudes (normalized to an area of 1) are plotted versus period for (E) Drinking patterns in the intact animal, (F) 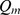 in the simulated intact animal, (G) Drinking patterns in the SCN-lesioned animal, (H) 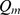 in the simulated SCN-lesioned animal. Note that the model uses a higher sampling rate than data. The data in panels (C), (E), and (G) are adapted from [43] and manually replotted here.

Although only fitted to the intact animal, the model reproduces realistic sleep/wake patterns for both intact and lesioned animals, as shown in **Figure 6**. With intact SCN, sleep is primarily monophasic, and free runs with a 25-h period (**Figure 6A**). With SCN lesions, the model correctly predicts that sleep is polyphasic (**Figure 6B**). Total daily sleep time is increased after SCN lesions, from 36% to 52% in data, and from 36% to 59% in simulations. Plotting percentage time awake as a function of circadian time shows a high degree of consistency between data (**Figure 6C**) and simulation (**Figure 6D**).

Spectral analysis of activity patterns (measured from drinking patterns for data, and 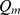 for model) shows a strong primary (fundamental) component at the circadian (∼25 h) period for intact animals, with smaller peaks at the second harmonic (∼12.5 h) period for data (**Figure 6E**) and simulation (**Figure 6F**). With SCN lesions, the circadian component is lost. The data show a spectral peak at an ultradian (shorter than 24 h) period of approximately 4 h (**Figure 6G**). The model similarly predicts a spectral peak at 5.2 h (**Figure 6H**). This ultradian sleep/wake cycle is due to the continued action of the sleep homeostat in the model; sleep pressure increases during wakefulness and decreases during sleep. The period of the ultradian cycle is therefore determined by the homeostatic time constant. The fact that the model's predicted 5.2-h period closely matches the 4-h period seen experimentally is remarkable, since the model parameters were not chosen to reproduce this feature. In fact, the homeostatic time constant was estimated based purely on sleep/wake patterns in the intact animal. This finding was also highly robust; increasing or decreasing the homeostatic time constant by 25% was found to produce ultradian periods of 5.8 h and 4.5 h, respectively. The prediction of ultradian sleep/wake cycles is therefore an emergent feature of the model and, to our knowledge, provides the first strong evidence for the sleep homeostatic process being the generator of this cycle in SCN-lesioned animals.

## Discussion

Experimental advances over the past decade have identified core mechanisms underlying mammalian sleep/wake regulation [38]. Consequently, mathematical modeling has become a powerful tool for relating overt behavior to physiology [40], [45], [46], [47], [48], [49], [50]. Here, we used a model of circadian signal modulation and masking by light to perform a novel analysis of how these two mechanisms influence the temporal organization of both rest/activity and sleep/wake patterns in a variety of species. Our findings demonstrate that these two mechanisms can together account for much of the observed interspecies variability in temporal niche. Furthermore, we showed that complete switching between diurnal and nocturnal phenotypes under LD and DD conditions requires simultaneous inversions in both masking and the circadian signal. Future studies are required to understand how these changes are coordinated and how the relevant environmental signals are transduced.

Understanding which neural mechanisms influence temporal niche has important evolutionary implications. It is postulated that the evolution of homeothermy enabled mammals to exploit a wider range of temporal niches [51]. However, it is not known precisely which components of sleep/wake and circadian circuitry were necessary to enable changes in temporal niche. Saper et al. [11] suggested that the multi-step neuronal pathway from SCN to sleep/wake switch may allow the SCN output signal to be flexibly modulated and integrated with other physiological signals. Our results support this hypothesis, showing that modulation of a single oscillator (i.e., SCN) output can yield a full spectrum of switchable phenotypes, from diurnal to nocturnal, as well as bimodal activity patterns. Integration with other signals, including food entrainment [52] and sleep homeostasis [40], could give rise to additional and more varied phenotypes.

We modeled circadian signal inversion at the SPZ, but alternative mechanisms for temporal niche switching may have evolved independently in other species. For example, the mole rat switches from diurnal to nocturnal behavior by changing how the SCN responds to light [53]. Similarly, a switch from nocturnal to diurnal behavior can be induced in mice by knocking out two genes that affect the retinal response to light [54]. Additionally, the phase relationships between arousal systems may be more complex than modeled here [55], due to different firing patterns within different parts of the SCN [56] and autonomous clock behavior within other neuronal populations [57]. In future, our model could be used to investigate these phenomena.

Many mammals display bimodal activity patterns. However, the physiological basis for this activity profile remains poorly understood. Pittendrigh and Daan proposed a two-oscillator model to account for this, in which each oscillator generates a single activity peak [58]. Such a mechanism appears to be present in Drosophila [59] and may underlie the splitting of activity into two distinct oscillations under LL conditions in some mammals [60]. While a two-oscillator system is one possible mechanism for generating bimodal activity patterns, our modeling shows that bimodal activity patterns can also emerge naturally from two (or more) competing outputs of a single oscillator in interaction with known sleep-regulatory systems.

Simulating SCN lesions, we reproduced the ∼4-h ultradian sleep/wake cycles of SCN-lesioned squirrel monkeys without making any special additions to the model or even adjusting any existing parameters, besides changing the amplitude of the SCN signal to zero in the SCN-lesioned case. The period of this sleep/wake cycle is determined by the value of the homeostatic time constant, which was estimated from sleep patterns in the intact animal. Our results thus provide quantitative evidence that ultradian sleep/wake cycles in SCN-lesioned animals can be explained parsimoniously by continued action of the sleep homeostatic process. This can be related to our recent finding that the ultradian REM/NREM sleep cycle in humans can also be generated by the very same sleep homeostatic process [61], with the intriguing implication that both polyphasic sleep/wake cycles and ultradian REM/NREM sleep cycles may have the same physiological basis.

Furthermore, our model reproduced the increase in average daily sleep duration caused by SCN lesions in the squirrel monkey. Whether the circadian signal is wake-promoting or sleep-promoting remains a matter of debate. While SCN lesions increase daily sleep duration in some species [43], they reduce daily sleep duration in others [42]. These conflicting findings have led to the notion that the SCN may actively promote both wake and sleep at different circadian phases, with the relative sleep/wake balance differing between species [62]. This dual output can be explained by the fact that SCN efferents employ multiple neurotransmitters [63] and this has been modeled explicitly elsewhere [46]. We allow for this in our model by incorporating a constant offset in the SCN signal to the SPZ; depending on its value, the SCN may be exclusively wake-promoting, exclusively sleep-promoting, or alternately wake-promoting and sleep-promoting at different circadian phases. After fitting parameters to the species simulated here, our model predicts that the circadian signal is exclusively wake-promoting in humans and squirrel monkeys, and alternately wake-promoting and sleep-promoting in degus and spider monkeys (see **Tables S1** and **S2** for all parameter values). These predictions may be tested experimentally in future work.

Going forward, one of the greatest challenges for the field will be reconciling the rest/activity patterns observed in the laboratory – on which we primarily focused here – with those observed in more natural settings [64], [65]. While work in the laboratory has provided a basic understanding of the salient physiological mechanisms and their key interactions, new methods, including models, will be needed to extend this knowledge to the inevitably more complex real-world scenarios. One advantage afforded by modeling is the ability to freely manipulate variables that would be extremely challenging to manipulate experimentally, and to then relate these variables to observable phenotypes. Modeling can also help to predict and target experimental protocols that will be most sensitive to the quantities of interest. In these respects, our approach is extremely powerful; our model successfully reproduces very diverse behaviors, generates new testable predictions, and yields unique insights into the underlying physiology.

## Methods

To simulate sleep/wake and rest/activity patterns, a combined model of the mammalian sleep/wake switch and circadian pacemaker was used [66]. The model's equations are briefly defined below, along with the metrics used to visualize the model's outputs and compare them to experimental data. For additional details, see **Text S1**.

### Mathematical Model

The model is based on a previously developed and validated model [40], [66], extended here to include the circadian relay system and masking by light. As shown in **Figure 1**, the model includes sleep-regulatory neuronal populations in the brainstem and hypothalamus, the circadian pacemaker and its system of relays to the sleep-regulatory system, and the effects of light on both the circadian pacemaker and the sleep-regulatory populations. The sleep-regulatory populations are the wake-promoting monoaminergic (MA) population and the sleep-promoting ventrolateral preoptic area (VLPO), which are mutually inhibitory and comprise the sleep/wake switch.

Mean cell body voltages, 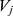, and mean firing rates, 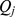, are defined for the MA (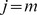) and VLPO (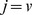) populations. The dynamics of these two populations are modeled by two coupled first-order differential equations [67],
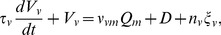
(1)

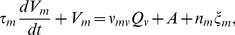
(2)where 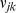 terms represent connection strength to population 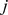 from 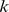, 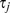 is a decay time constant, 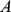 is input from cholinergic and other sources, 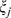 is additive Gaussian-distributed white noise, and 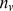 and 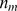 are constants.

Inputs to the sleep-regulatory VLPO and MA from other sources are respectively represented by
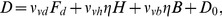
(3)

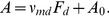
(4)The term 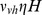 is the homeostatic sleep drive, with 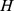 obeying the first-order differential equation
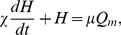
(5)where 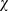 is a time constant and 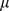 is constant. During wake, 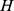 saturates approximately exponentially to 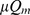. During sleep, 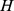 decays approximately exponentially to 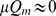.

The 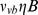 term in Eq. 3 represents the direct (masking) effects of light on the VLPO, with excitatory (sleep-promoting) input (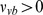) corresponding to negative masking and inhibitory (wake-promoting) input (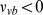) corresponding to positive masking. The relationship between 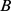 and environmental light is described below.

The 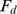 term in Eqs. 3 and 4 represents relayed circadian output via the DMH. It is defined by the linear equation
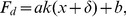
(6)where 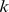, 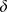, and 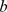 are constants, 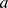 represents modulation of SCN output by the SPZ, and 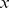 represents the output of the master circadian pacemaker, the SCN.

The dynamics of 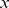 are modeled by a modified van der Pol oscillator [68],
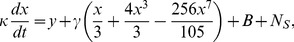
(7)

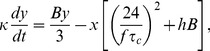
(8)where 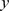 is a complementary variable with no physiological interpretation. The 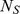 term represents the effects of non-photic stimuli on the circadian pacemaker. This is modeled by
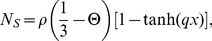
(9)where 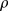 and 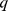 are constants, and 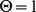 during wake and 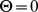 during sleep. The non-photic effects are very small compared to the effects of light [69].

The effect of environmental light, 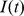, on retinal photoreceptors is modeled [70]. Photoreceptors are assumed to be in either a ready state or an activated state. In the absence of light, photoreceptors move into the ready state at a rate 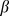. The arrival of photons converts ready receptors to an activated state at rate
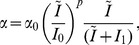
(10)where 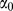, 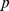, 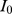, and 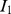 are constants. This functional form was chosen previously to match empirical data [69]. 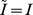 when awake (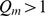 s^−1^) and 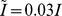 during sleep, to simulate the effects of eye closure. The fraction 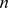 of photoreceptors that are activated is modeled by
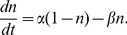
(11)


Activation of photoreceptors results in a signal to the SCN proportional to the rate of activation: 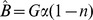, with 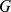 constant. To account for the fact that the SCN is more sensitive to light at certain circadian phases, the signal is also modulated by circadian phase, 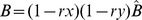, where 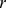 is constant [71].

Physiological ranges on most of the model's parameter values have previously been estimated for humans and 16 other mammalian species [40], [67], [72]. Parameter values for species modeled here (spider monkeys, degus, and squirrel monkeys) were estimated based on the taxonomy of each species and fitting to experimental data. All parameter values are given in **Tables S1** and **S2**, and the methods of parameter estimation are presented in the **Text S1**.

### Model Outputs

The model described above generates time series for all its variables when provided with a light input function, a set of parameter values, and initial conditions. Using this model, we simulated different species and experimental conditions, including the experiments of Edgar et al. [43] and Kas and Edgar [9]

As in previous work [40], the firing rate of the MA population, 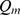, is taken as a proxy for arousal state, because MA firing rate correlates closely with arousal [39]. When 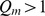 s^−1^, the model is considered to be awake, and when 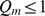 s^−1^, the model is considered to be asleep.

Here, we also compare the model predictions with rest/activity data, which are usually time-averaged (typically in activity counts per unit time). We were therefore motivated to develop a model proxy for activity. For this, we time averaged 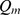 in windows of width 10 min.

## Supporting Information

Figure S1
**Daily patterns of monoaminergic firing rates under DD conditions.** Simulations of MA firing rate, 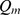, are shown across a 24-h time interval for different values of 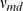. Positive (excitatory) 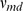 results in an activity pattern that peaks near the middle of the waking period. Negative (inhibitory) 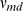 results in a bimodal activity pattern.(TIF)

Figure S2
**Degu simulations with different activity thresholds.** Simulations of the same degu switching protocol used in **Figure 5B**, using thresholds of (A) 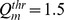 s^−1^, (B) 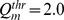 s^−1^, (C) 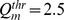 s^−1^ for bouts of activity.(TIF)

Table S1 **The full set of model parameters and their estimated values and units for humans.** Parameters are grouped by type. Values are a combination of those estimated previously [1] and those estimated here. With the exception of parameters listed in **Table S2**, these values are used for all model simulations.
(DOC)

Table S2 **Estimated values of parameter for each species.** Parameter values for each species are derived from constraints in **Text S1**. All other parameter values are kept constant for all species, with values given in previous work [1] and **Table S1**. Corresponding Figures in the paper are listed for each parameter set.
(DOC)

Text S1 **Detailed description of the mathematical model, its implementation, and the methods of parameter constraint.** This supplementary text file complements the **Methods** section by providing additional information about the model and its parameter values.
(PDF)
