# Supplementary figures and images for: Mammalian Rest/Activity Patterns Explained by Physiologically Based Modeling
Source: PLoS Comput Biol. 2013 Sep 5;9(9):e1003213. doi: 10.1371/journal.pcbi.1003213 (PMC3764015; doi:10.1371/journal.pcbi.1003213)

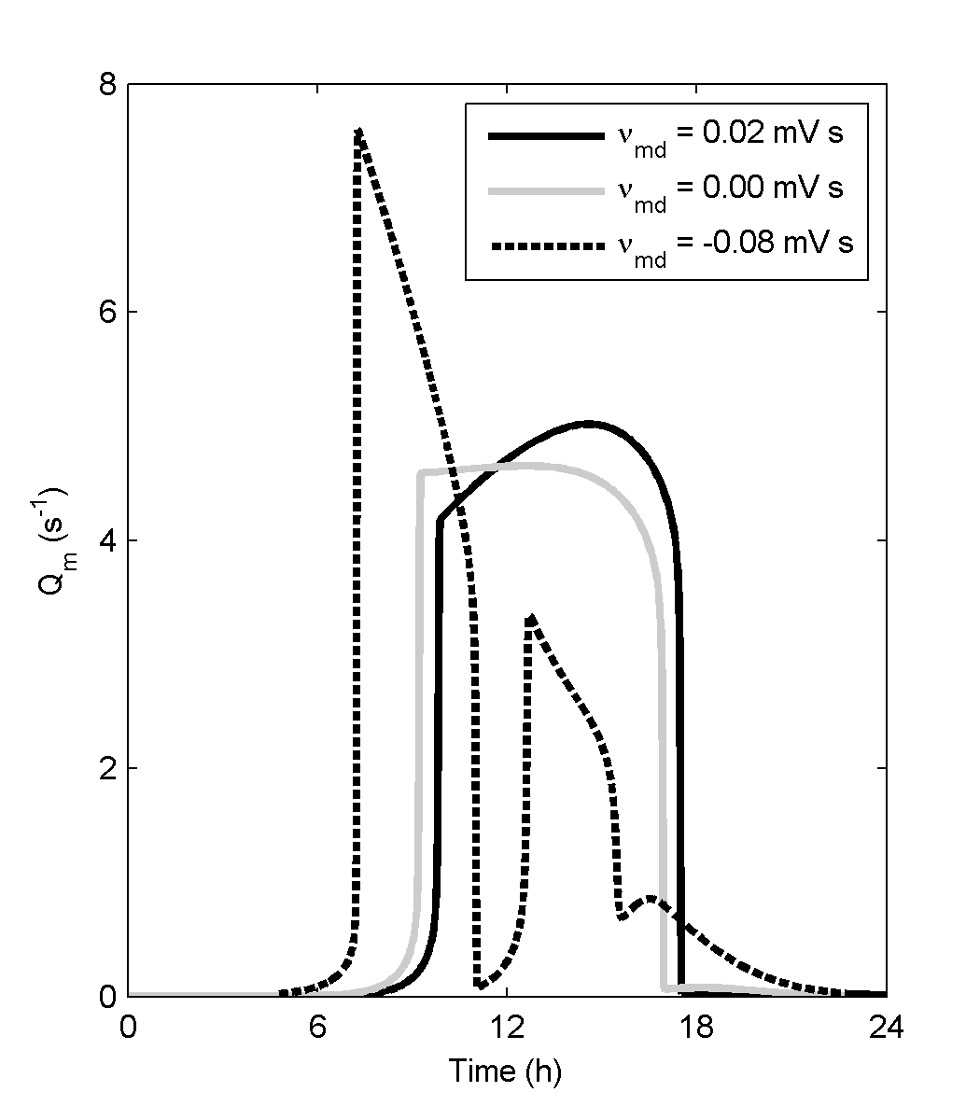

Supplement: Figure S1 — Daily patterns of monoaminergic firing rates under DD conditions. Simulations of MA firing rate, , are shown across a 24-h time interval for different values of . Positive (excitatory) results in an activity pattern that peaks near the middle of the waking period. Negative (inhibitory) results in a bimodal activity pattern. (TIF) [file pcbi.1003213.s001.tif]

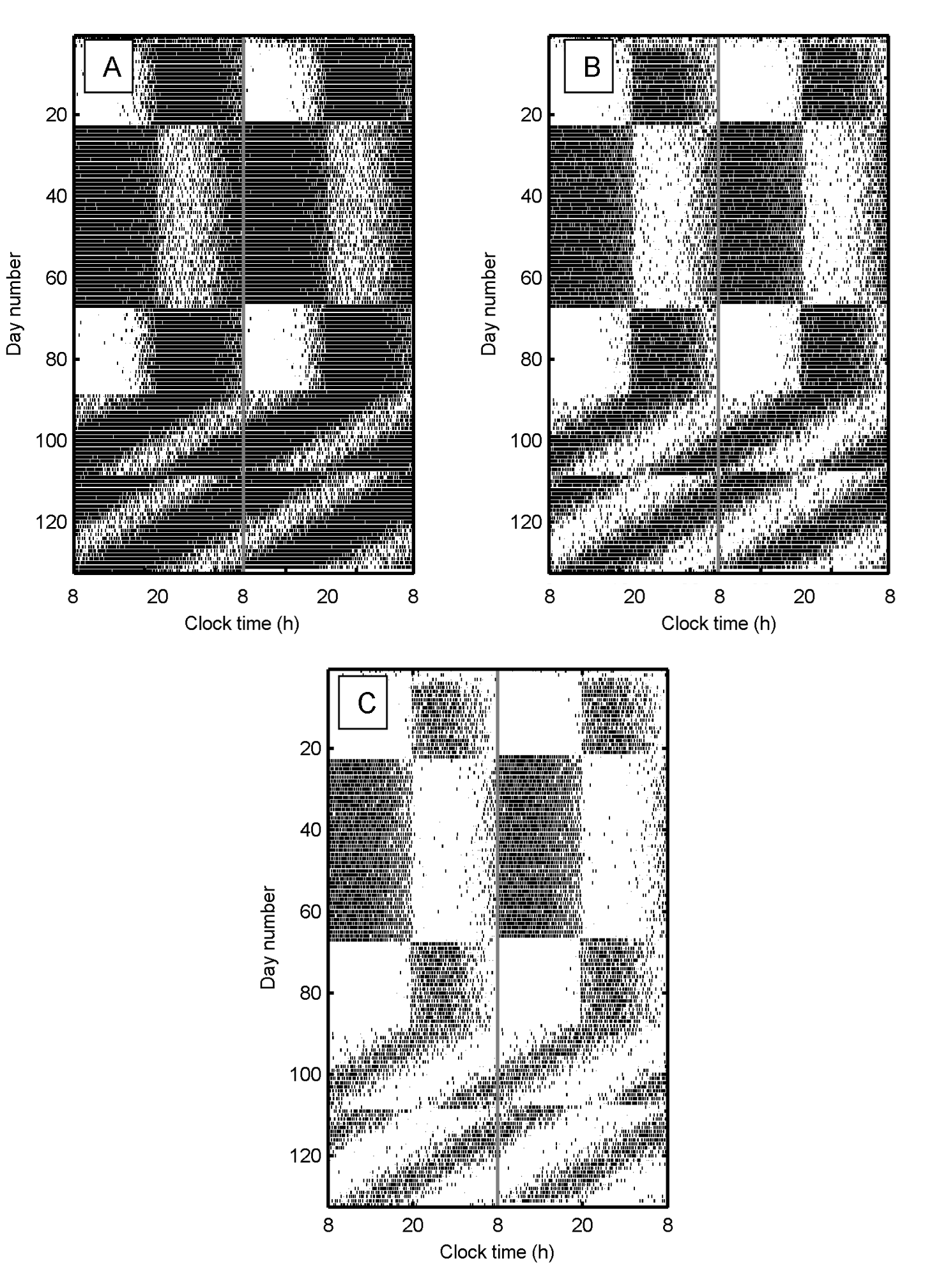

Supplement: Figure S2 — Degu simulations with different activity thresholds. Simulations of the same degu switching protocol used in Figure 5B , using thresholds of (A) s−1, (B) s−1, (C) s−1 for bouts of activity. (TIF) [file pcbi.1003213.s002.tif]
