# Supplementary material for: Mammalian Rest/Activity Patterns Explained by Physiologically Based Modeling
Source: PLoS Comput Biol. 2013 Sep 5;9(9):e1003213. doi: 10.1371/journal.pcbi.1003213 (PMC3764015; doi:10.1371/journal.pcbi.1003213)
Supplement: Table S1 — The full set of model parameters and their estimated values and units for humans. Parameters are grouped by type. Values are a combination of those estimated previously [1] and those estimated here. With the exception of parameters listed in Table S2, these values are used for all model simulations. (DOC) [file pcbi.1003213.s003.doc]

| Parameter | Value | Unit | Parameter | Value | Unit |
| --- | --- | --- | --- | --- | --- |
| Retinal Processing | | | Relay Connections | | |
|  | 6 | h^-1^ |  | -0.17 | mV s |
|  | 9500 | lux |  | 0.01 | mV s |
|  | 100 | lux | Neuronal Inputs | | |
|  | 0.5 | - |  | -880 | mV s |
|  | 0.4 | h^-1^ |  | 1 | s^-1^ |
| Photic Drive | | |  | 6 | s |
|  | 37 | - |  | 3.2 | mV |
|  | 0.4 | - |  | 1.3 | mV |
|  | 0.55 | - |  | -4.8 | mV |
| Non-photic Drive | | | Sleep Homeostatic Drive | | |
|  | 10 | - |  | 100 | s^-1^ |
|  | 0.032 | - |  | 10 | mV |
| Circadian Pacemaker | | |  | 3 | mV s |
|  |  | h | Sigmoidal Firing Rate Function | | |
|  | 0.99729 | - |  | 100 | s^-1^ |
|  | 24.1 | h |  | 10 | mV |
|  | 0.13 | - |  | 3 | mV |
| Circadian Relay | | | Neuronal Populations | | |
|  | 2.8 | - |  | -2.1 | mV s |
|  | 1 | - |  | -1.8 | mV s |
|  | 17 | s^-1^ |  | 10 | s |
|  | 4.8 | s^-1^ |  | 10 | s |
