# Supplementary material for: Mammalian Rest/Activity Patterns Explained by Physiologically Based Modeling
Source: PLoS Comput Biol. 2013 Sep 5;9(9):e1003213. doi: 10.1371/journal.pcbi.1003213 (PMC3764015; doi:10.1371/journal.pcbi.1003213)
Supplement: Table S2 — Estimated values of parameter for each species. Parameter values for each species are derived from constraints in Text S1. All other parameter values are kept constant for all species, with values given in previous work [1] and Table S1. Corresponding Figures in the paper are listed for each parameter set. (DOC) [file pcbi.1003213.s004.doc]

| Species |  |  | (h) | (lux) | (mV s) | (h) | (mV s) |  |
| --- | --- | --- | --- | --- | --- | --- | --- | --- |
| Human | 1 | 2.8 | 45 | 100 | 0.01 | 24.2 | -880 | 37 |
| Rodent Example  (Figure 2) | -1 | 0.4 | 0.3 | 0.04 | 0 | 23.9 | 0 | 37 |
| Spider Monkey  (Figure 3) | 1 | -0.7 | 22 | 10 | -0.08 | 24.2 | 0 | 37 |
| Degu  (Figure 4) | 1 | 0.0 | 0.3 | 0.04 | 0.01 | 23.0 | -440 | 74 |
| Squirrel Monkey  (Figure 5) | 1 | 2.9 | 22 | 10 | 0.01 | 25.0 | -880 | 37 |
