# Supplementary material for: Mammalian Rest/Activity Patterns Explained by Physiologically Based Modeling
Source: PLoS Comput Biol. 2013 Sep 5;9(9):e1003213. doi: 10.1371/journal.pcbi.1003213 (PMC3764015; doi:10.1371/journal.pcbi.1003213)
Supplement: Text S1 — Detailed description of the mathematical model, its implementation, and the methods of parameter constraint. This supplementary text file complements the Methods section by providing additional information about the model and its parameter values. (PDF) [file pcbi.1003213.s005.pdf]

Text S1

In this section, we describe our mathematical model of mammalian sleep and circadian physiology. We then describe how the model parameter values were estimated, introduce the model proxy for activity, and describe the methods used to generate **Figures 5** and **6** in the main manuscript.

Model

We previously developed a combined mathematical model of the mammalian sleep/wake switch and circadian pacemaker [1]. All of the parameters of this model have been rigorously constrained by fitting to experimental data [2,3,4,5,6].

In this work, we extended the model to include two new features, shown in **Figure 1**:

(i) The neuronal relay system that conveys SCN output to the sleep/wake switch.

(ii) The direct (masking) effect of light on the VLPO.

These extensions added new parameters to the model. Below, we describe the rigorous procedures used to constrain these new parameters.

Circadian Model

Our circadian model includes the circadian pacemaker, retinal processing of photic stimuli, and the effects of photic and non-photic stimuli on the pacemaker [3]. As in previous work [7], we assumed that environmental light, $I(t)$, is gated by arousal state to yield the light reaching the retina:

$$\tilde{I}(t) = \Omega I(t) \qquad \text{(Eq. S1)}$$

where Ω is the fraction of environmental light that reaches the retina. This functional form is used to simulate an animal that is free to choose its sleep and wake times but is unable to modify environmental light levels. In the 400-600 nm range, approximately 3% of light is transmitted by the eyelids [8,9,10]. We therefore used $\Omega = 1$ during wake and $\Omega = 0.03$ during sleep. Masking effects are mediated via the same retinal pathway and thus share the same action spectrum and sleep/wake dependence. This is a conservative reduction in retinal light during sleep, as other light-avoiding behaviors such as postural changes and light avoiding/dark seeking behaviors (e.g., covering eyes with paws) may further reduce retinal light exposure. Simulations using $\Omega = 0$ during sleep (not shown) yielded very similar results to simulations using $\Omega = 0.03$.

Retinal photoreceptors are assumed to be in either a ready state or an activated state. In the absence of light, retinal photoreceptors are considered to be in a ready state. The arrival of photons converts ready receptors to an activated state at rate

$$\alpha = \alpha_0 \left(\frac{\tilde{I}}{I_0} \right)^p \frac{\tilde{I}}{(\tilde{I} + I_1)}, \qquad \text{(Eq. S2)}$$

where α_0, p, I_0, and I_1 are constants, with this functional form being chosen previously to match empirical data [3]. Activated photoreceptors are converted back to the ready state at a constant rate β [2]. The fraction n of photoreceptors that are activated is thus modeled by

$$\frac{dn}{dt} = \alpha(1-n) - \beta n \,. \qquad \text{(Eq. S3)}$$

Activation of photoreceptors results in a photic drive to the pacemaker that is assumed to be proportional to the rate of activation: $\hat{B} = G\alpha(1-n)$, with G constant. To account for the fact that the circadian pacemaker is more sensitive to light at certain circadian phases [11], the photic input to the pacemaker is also modulated by circadian phase, yielding $B = (1-rx)(1-ry)\hat{B}$, where x and y are the variables of the circadian pacemaker, and $r = 0.4$ is used to match experimental data [2].

The circadian pacemaker is modeled by two first order differential equations, originally based on a Van der Pol oscillator [12],

$$\kappa \frac{dx}{dt} = y + \gamma\left(\frac{x}{3} + \frac{4x^3}{3} - \frac{256x^7}{105}\right) + B + N_S \,, \qquad \text{(Eq. S4)}$$

$$\kappa \frac{dy}{dt} = \frac{By}{3} - x\left[\left(\frac{24}{f\tau_c}\right)^2 + hB\right], \qquad \text{(Eq. S5)}$$

with x representing SCN activity and y being a complementary variable. The parameter h determines the photic drive strength, τ_c is intrinsic period, f is a term included to fix the free-running period to τ_c under total darkness (DD) conditions, γ determines the oscillator's stiffness, and $\kappa = (12/\pi)$ h [2]. The non-photic drive, N_S, models effects of sleep/wake patterns on the circadian clock (wake being signified by periods of high MA activity here) and is given by

$$N_S = \rho\left(\frac{1}{3} - \Theta\right)[1 - \tanh(qx)], \qquad \text{(Eq. S6)}$$

where ρ and q are constants, and $\Theta = 1$ during wake and $\Theta = 0$ during sleep [3].

All parameters of this model have been estimated previously using human experimental data, including phase response curves for light, dose response curves for the effects of light on circadian phase and melatonin expression, and non-photic entrainment in a blind individual [2,3]. The effects of the non-photic drive are much weaker than the effects of light and had very little impact on the results presented in this paper.

Due to the biological similarity of the circadian pacemaker between mammalian species [13,14], we used the same parameter values for all species, except where there was clear evidence to show that a particular aspect of circadian function differs, as explained below. Our primary objective here was to show that the same model can parsimoniously account for phenotypes displayed by a range of species under various experimental conditions. In future, fitting other parameters for particular species could improve model predictions. For example, phase and masking responses to non-photic stimuli may differ between diurnal and nocturnal species [15], which we did not attempt to reproduce here.

Circadian Relay System

To model the relay of circadian signals from the SCN to the sleep/wake switch, we included outputs of nuclei along this pathway. These nuclei include the SCN, SPZ and DMH, the outputs of which we denote by F_s, F_z, and F_d, respectively. Since

the circadian relay involves both excitatory and inhibitory neurotransmitters [16], we allowed these outputs to be both positive and negative, where positive represents net excitatory output and negative represents net inhibitory output. Because the sign of excitation/inhibition is incorporated into these outputs, they are not conventional firing rates, which are typically restricted to positive values. We treat the outputs as linear functions of firing rate and therefore use units of s^{-1}. In future, we could explicitly model multiple neurotransmitter outputs for each neuronal population, as Fleshner et al. did for the SCN [17].

During entrainment or free-running, x is approximately sinusoidal, ranging between -1 and 1. Multi-unit recordings in rats [18], chipmunks [19] and guinea pigs [20] have shown that SCN output, F_s, is also approximately sinusoidal. Thus, we assumed that F_s is a linear function of x,

$$F_s = s(x+\delta), \qquad \text{(Eq. S7)}$$

where s and δ are constants. For $\delta > 1$, F_s is always positive, while for $\delta < -1$, F_s is always negative. To account for the fact that the SCN makes use of multiple neurotransmitters, and thus may actively promote wake at some circadian phases and actively promote sleep at others [16], we did not restrict the value of δ.

Experimental evidence suggests that inversion of the circadian signal in nocturnal animals occurs at the level of the SPZ [21,22]. Since firing rates are approximately sinusoidal in both the SCN and adjacent hypothalamic regions [20], we assumed a linear relationship between SCN output and SPZ output: $F_z = aF_s + c = as(x+\delta) + c$,

where c is a constant and a is a multiplicative factor that modulates the circadian signal. We used fixed values of $a = 1$ for diurnal animals and $a = -1$ for nocturnal animals. In simulating animals that switch temporal niche, we allowed a to change its value, as described below.

Similarly, we assumed a linear relationship between F_z and F_d, $F_d = fF_z + g = afs(x + \delta) + (fc + g)$, which can be simplified to

$$F_d = ak(x + \delta) + b \text{ ,} \qquad \text{(Eq. S8)}$$

where $k = fs$ and $b = fc + g$ are constants. Note that below we fitted only values for k and b, as the absorbed constants (f , s , c , g) are redundant in terms of the model's sleep/wake and rest/activity predictions. Nevertheless, these absorbed parameters are important for formulating the model and could potentially be estimated in future by direct comparison to multi-unit recordings from each of the relay nuclei.

Sleep/Wake Switch Model

Phillips and Robinson previously developed a neural mass model of the sleep/wake switch [4]. The model includes the mutually inhibitory MA and VLPO populations, with inputs from circadian relays and the sleep homeostat, as shown in **Figure 1**. The model distinguishes wake and sleep, but not stages of sleep. The MA group receives input from cholinergic and other sources. All parameters of this model have been constrained rigorously in previous work by fitting to a wide range of behavioral and physiological data [4,5,6].

For each population, $j = v, m$, where v is VLPO and m is MA, we defined a mean cell body potential, $V_j(t)$, relative to resting, and a mean firing rate, $Q_j(t)$. We assumed spatial homogeneity of each population and neglected propagation delays due to their small spatial extent and relative proximity. The firing rate, Q_j, was approximated by a sigmoid function of V_j,

$$Q_j(t) = S[V_j(t)] = \frac{Q_{\max}}{1 + \exp[(\theta - V_j)/\sigma']}, \qquad \text{(Eq. S9)}$$

where $Q_{\max}$ is the maximum possible firing rate, θ is the mean firing threshold relative to resting, and σ' determines the spread of the sigmoid [23].

The differential equations defining the model are

$$\tau_v \frac{dV_v}{dt} + V_v = \nu_{vm} Q_m + D + n_v \xi_v, \qquad \text{(Eq. S10)}$$

$$\tau_m \frac{dV_m}{dt} + V_m = \nu_{mv} Q_v + A + n_m \xi_m, \qquad \text{(Eq. S11)}$$

where ν_{jk} terms represent connection strength to population j from k, τ_j is the characteristic decay time for group j neuromodulators, A is input from cholinergic and other sources, ξ_j is additive Gaussian-distributed white noise of mean 0 and standard deviation 1, and n_v and n_m are constants that control the standard deviation of the added external noise.

Without noise in the model, rest/activity and sleep/wake patterns are identical on each day. For monophasic sleepers this means a completely consolidated block of daily sleep, as shown previously [4]. In reality, rest/activity and sleep/wake patterns are intrinsically variable. We simulated these features by adding Gaussian-distributed white noise to the model. Here, we set $n_v = n_m = n$ for simplicity, i.e., in the absence of any evidence either way, we set the external noise standard deviations to the VLPO and the MA to be equal. Noise was generated independently for each population in each simulation.

The total drive to the VLPO from sources other than the MA and noise is

$$D = \nu_{vd} F_d + \nu_{vh} \eta H + \nu_{vb} \eta B + D_0 , \qquad \text{(Eq. S12)}$$

where $\nu_{vd} F_d$ represents input from the DMH, $\nu_{vh} \eta H$ is the sleep homeostatic drive, $\nu_{vb} \eta B$ represents the direct retinal effect of light on the VLPO, and D_0 is a constant offset representing net input to the VLPO from all other sources. The parameter $\eta = 1$ s^{-1} is a conversion factor to give ν_{vb} and ν_{vh} the same dimensionality of mV s as all other ν_{jk} terms in the model.

In Eq. S12, the constant terms can be grouped to give the term $\nu_{vd} (ak\delta + b) + D_0$, using the definition of F_d from Eq. S8. The value of this term mostly controls the total amount of daily sleep, with relatively little effect on other dynamics. Its value can therefore be fitted for different species. However, additional constraints exist, which we use for parameter fitting below. First, SCN lesions ($a = 0$) result in

approximately 50% of time spent in sleep, independent of species [24,25,26,27]. Therefore $\nu_{vd} b + D_0$ must be approximately constant between species. In addition, we can estimate the value of D_0 from DMH lesions ($\nu_{vd} b = 0$). We are therefore left with the circadian baseline offset parameter, δ, for fitting daily sleep duration. We note that in previous work, the circadian baseline offset parameter was called c_0 and the mathematical form of the input to the VLPO was $D = \nu_{vc} x + \nu_{vc} c_0 + \nu_{vh} H$ [6]. By equating constant terms between the previous and current definitions of D, we can express c_0 as a linear function of δ: $c_0 = \left[\nu_{vd}\left(ak\delta + b\right) + D_0\right]/\nu_{vc}$. For the parameter values derived below, this simplifies to $c_0 \approx \delta + 1.9$.

The total drive to the MA from sources other than the VLPO and noise is

$$A = \nu_{md} F_d + A_0, \qquad \text{(Eq. S13)}$$

where A_0 is a constant that includes the combined time-averaged effects of cholinergic, orexinergic, and other sources. The term $\nu_{md} F_d$ represents the circadian signal relayed via the DMH/LHA pathway, assuming it is a linear function of F_d and absorbing any constant offset into A_0.

The sleep homeostatic drive, H, represents the amount of some somnogen (sleep-promoting substance) that increases during wake and decreases during sleep. One candidate for this somnogen is extracellular basal forebrain adenosine [28], although we note that the same functional form is equally applicable to other sleep-promoting factors which accumulate during wake and are cleared during

sleep, such as cytokines [29]. Production is approximated by a linear function of Q_m [5], because MA activity correlates with arousal [30]. Clearance is assumed to be proportional to concentration, yielding

$$\chi \frac{dH}{dt} + H = \mu Q_m \,, \qquad \text{(Eq. S14)}$$

where χ is the characteristic time for somnogen accumulation and clearance, and μ is constant.

Parameter Estimation

The parameters of the circadian and sleep/wake switch models have previously been constrained rigorously for humans [3,4,5,31], and values can be found in **Table S1**. However, the addition of the circadian relay system, light's effect on the VLPO, and noise introduced 8 new parameters: a, k, δ, b, ν_{vd}, ν_{md}, ν_{vb}, and n. We thus needed to estimate realistic values for these parameters while preserving the previous behavior of the model. We did not allow any free fitting of the new parameters; they are all tightly constrained by physiology.

We began by fixing all old parameters (i.e., parameters that were previously included in the model) at their previously published values. In general, we worked from a principle of interspecies similarity, motivated by our previous finding that the sleep/wake switch model can reproduce the sleep patterns of a wide variety of mammals through changes in just two parameters: the circadian baseline offset, δ (for fitting sleep duration, as explained above), and the homeostatic time constant,

χ [6]. Since light, orexin, and noise all slightly affect the model's daily sleep duration, we left calibration of the parameter δ for each species until last; this parameter modifies daily sleep duration in both diurnal and nocturnal animals with relatively little effect on other dynamics [6].

To simplify the task of parameter constraint, we explored reduced parameter spaces under conditions where other unconstrained parameters have relatively little effect. We thus began by constraining the parameters associated with the circadian relay system by restricting our focus to DD. We then introduced the effects of light, followed by the role of the DMH/LHA pathway in DD. Finally, we introduced noise and fitted the model to individual species.

Relay System

Firing rates for DMH neurons have yet to be assessed *in vivo*. Nevertheless, we were able to estimate values of a, k, δ, and b based on how these parameters affect the model dynamics. SCN lesions have been variably reported to increase, decrease, or not affect total sleep duration in different species [24,25,26,27]. However, the common theme to these experiments is that SCN-lesioned animals sleep very close to 50% of the time; the same is true of animals with SPZ lesions [32]. Furthermore, it has been found that DMH lesions in rats increase daily sleep duration in DD by 0.9±0.6 h (from 12.1±0.4 h to 13.0±0.2 h) [33]. We used these results to infer parameter values by simulating SCN and DMH lesions in rats.

We simulated SCN lesions in DD conditions by setting $a = 0$ and $B = 0$, whence Eq. S12 reduces to $D = (\nu_{vd} b + D_0) + \nu_{vh} H$. To produce a sleep/wake cycle with 11.5-12.5 h sleep (i.e., approximately 50% sleep), the constant term in brackets is required to satisfy –8.1 mV $< \nu_{vd} b + D_0 < -5.0$ mV for 0.1 h < χ < 50 h, based on simulations across this parameter range. Setting $\chi = 0.3$ h to simulate the polyphasic sleep of rats [6], we found $\nu_{vd} b + D_0 = -5.6$ mV yields a 12.0 h daily sleep duration.

We simulated DMH lesions in DD conditions by setting $\nu_{vd} = 0$ and $B = 0$, whence Eq. S12 reduces to $D = D_0 + \nu_{vh} H$. Again using $\chi = 0.3$ h to simulate rats, we found that $D_0 = -4.8$ mV yields a sleep duration of 13.0 h, matching data [33]. This implies that $\nu_{vd} b = -0.8$ mV, where the parameter b can be thought of as representing the DMH output in an animal with an SCN lesion ($a = 0$). In the absence of *in vivo* studies of typical DMH neuronal firing rates, we used results from an *in vitro* whole-cell patch-clamp study [34] to estimate plausible parameter values. We used the resting firing rate of DMH cells that project to sites other than the paraventricular nucleus to set $b = 4.8$ s^{-1}, thus yielding $\nu_{vd} = -0.17$ mV s.

In an intact animal that is diurnal or nocturnal ($a = \pm 1$), the range of DMH output across a full circadian cycle ($-1 \le x \le 1$) is determined from Eq. S7 to be $[k(1+\delta) + b] - [k(-1+\delta) + b] = 2k$. The amplitude of the input to the VLPO is thus

$|k\nu_{vd}|$. In previous work, the amplitude was determined to be $-\nu_{vc} = 2.9$ mV [5]. Thus, for consistency we used

$$k = \frac{\nu_{vc}}{\nu_{vd}} = 17\ \text{s}^{-1}. \qquad \text{(Eq. S15)}$$

Sensitivity to Light

The parameters of Eq. S2 have previously been estimated for humans, but experimental findings suggest that there are significant interspecies differences in light sensitivity. In humans, it has been shown that the half maximum response to light after sensitization to darkness, I_1, is at approximately 100 lux [35]. However, studies in hamsters find a half maximum response at approximately 0.5 mW/m^2 [36]. Assuming a luminous efficiency of no more than 10% for fluorescent bulbs [37] yields a conversion of no more than 0.07 lux per mW/m^2. Thus $I_1 \leq 0.04$ lux for hamsters. We used the value $I_1 = 0.04$ lux as an estimate for all rodents. We note that different physiological responses to light (e.g., melatonin suppression, phase resetting) could have different thresholds in the same animal, which we did not attempt to model here.

Masking by Light

To estimate the strength of the retinal projection to the VLPO in mammals, we used sleep duration for Sprague-Dawley rats kept in constant dark (DD) and light/dark (LD) cycle conditions. Rats sleep 48.0±1.0% of the time in DD conditions, and 49.7±1.4% of the time on a 24-h LD cycle with 12 hours of 30 lux,

corresponding to negative masking, as expected for a nocturnal animal [38]. We fitted the model to the mean values. To simulate rat sleep, we used $\chi = 0.3$ h, $a = -1$, and $\delta = 0.142$ to reproduce 48.0% sleep under DD conditions. Noise and orexin effects were omitted to examine the effect of masking in isolation. We then varied the parameter ν_{vb} under LD conditions, measuring the average sleep duration across a 40 day simulation, after a 20 day entrainment period to avoid transients. Fitting the model to reproduce 49.7% sleep under LD conditions yielded $\nu_{vb} = 880$ mV s.

Orexinergic Pathway

The circadian signal is projected to the MA group via two main pathways: direct input from the SCN to the locus coeruleus [39], and from the DMH via orexinergic neurons in the LHA [33]. Since the latter connection is considered to be the dominant circadian relay [40], we omitted the former. To test the effects of the DMH/LHA relay on daily activity patterns, we varied the value of ν_{md}. We simulated monophasic diurnal sleep patterns using $\chi = 22$ h, $a = 1$, and $\delta = -0.7$, and used DD conditions to examine the effects of this pathway independent of masking. No noise was yet included in the model, so the predictions were deterministic. We assumed that $|\nu_{vd}| > |\nu_{md}|$, as our previous work has used $\nu_{md} = 0$. This means that the DMH-VLPO relay is dominant over the DMH-LHA relay.

As shown in **Figure S1**, $\nu_{md} > 0$ results in a slight enhancement of waking MA firing rates in the second half of the waking day. This is due to the DMH-VLPO and

DMH-LHA relays acting in concert to promote wake: $\nu_{md} > 0$ promotes MA activity, while $\nu_{vd} < 0$ inhibits VLPO activity. However, when $\nu_{md} < 0$, the relays are in opposition, resulting in a slight suppression of MA activity near the middle of wakefulness. For $\nu_{md} = -0.08$ mV s, activity patterns are diurnal and bimodal.

Noise Amplitude

We simulated variability in the model by adding Gaussian-distributed white noise to Eqs. S10 and S11, with standard deviation determined by the parameter n. To estimate the value of n we used the wakefulness distribution across circadian phase reported for squirrel monkeys by Edgar et al. [25], shown in **Figure 6C**. During the inactive phase, squirrel monkeys were found to be awake ~10-20% of the time. To reproduce a similar level of night time wakefulness without disrupting daytime wakefulness, we used $n = 1.3\,\mathrm{mV\,s}^{-1/2}.\Delta T^{1/2}$, where ΔT is the size of the time step used for numerical integration. Here, we used $\Delta T = 6$ s for all simulations (using a fourth order Runge-Kutta algorithm in Matlab), so $n = 3.2\,\mathrm{mV}$.

Species

In this paper, we simulated the rest/activity and sleep/wake patterns of several species. We varied as few parameter values as possible between species, on the basis of their broadly similar physiology. As described above, 8 parameters needed to be estimated. Each parameter was estimated based on physiological and behavioral constraints.

(i) The parameter a multiplicatively scales the circadian signal, simulating modulation at the level of the SPZ. In diurnal species we used $a = 1$ and in nocturnal species we used $a = -1$.

(ii) The circadian baseline offset parameter, δ, controls sleep duration with relatively little effect on other dynamics. We thus used it to fit daily sleep duration, as in previous work [6].

(iii) The homeostatic time constant, χ, determines the rate of cycling between wake and sleep, and has been estimated for multiple species [6]. Based on these previous findings, we used $\chi = 0.3$ h for rodents to generate polyphasic sleep patterns. For the squirrel monkey, we used $\chi = 22$ h to produce primarily monophasic sleep patterns similar to experimental data [41]. We used the same value for the spider monkey.

(iv) The intensity required for half maximal response to light, I_1, was determined above to be ~0.04 lux for hamsters. We used this value for all rodents. For humans it is known to be ~100 lux [35]. Experimental evidence shows that squirrel monkeys are relatively sensitive to light [42], but the dose response curve has not been quantified. We thus chose an intermediate value of $I_1 = 10$ lux for squirrel monkeys and used the same value for spider monkeys. Note that although this parameter ranges across ~3 orders of magnitude, its value only significantly affects sensitivity to low light levels, where the effects of light are already

relatively weak. For an environmental light intensity of 30 lux (the lowest non-zero light level modeled here), $I_1 = 0.04$ lux results in a response 1.3 times stronger than $I_1 = 10$ lux with eyes open, and 20 times stronger with eyes closed.

(v) The orexin to MA connection strength, ν_{md}, has not been directly measured, but it is believed to be excitatory in some species [33,40]. To achieve similar model dynamics to those explored previously, we chose a conservative value of 0.01 mV s (which is small compared to ν_{vd}) for the species simulated here, with the exception of the spider monkey, where we used $\nu_{md} = -0.09$ mV s to show that an inhibitory connection can generate a bimodal activity profile (**Figure 3B**).

(vi) The intrinsic circadian period, τ_c, has been estimated experimentally for each species modeled here. In humans under forced desynchrony, the circadian period is 24.1 h [43], so we used $\tau_c = 24.1$ h. For degus, the free-running period under DD conditions is 23.0 h [44], so we used $\tau_c = 23.0$ h. For spider monkeys and squirrel monkeys, free-running periods have been assessed under constant light (LL) conditions. In these cases we cannot directly use the observed period as the value of τ_c, because constant light can alter the observed circadian period [45]. We therefore found the value of τ_c that resulted in the observed free-running period under simulated LL. For spider monkeys observed under

LL conditions (107 lux), the free-running period is 23.9 h [46]. We used $\tau_c = 24.2$ h to reproduce this free-running period under LL. For squirrel monkeys observed under LL conditions (500 lux), the free-running period is 25.0 h [25]. We used $\tau_c = 25.0$ h to reproduce this free-running period under LL. For our rodent example, we used a value of $\tau_c = 23.9$ h [45].

(vii) The retina to VLPO connection strength, ν_{vb}, was estimated based on the above fit to Sprague-Dawley rat data [38]. For negative masking we used $\nu_{vb} = 880$ mV s and for positive masking we simply inverted the effect of light on the VLPO by using $\nu_{vb} = -880$ mV s. It has been proposed that interneurons could achieve such an inversion [47], although this has yet to be confirmed experimentally.

(viii) The strength of the retinal signal to the SCN, G, has previously been calibrated for humans. We used this value for all species, with the exception of the degu. Model simulations of degu sleep/wake patterns were found not to entrain to a 24-h LD cycle with 12 h of 30 lux. This is contradicted by experimental data [44], suggesting that degus achieve larger phase responses than human-fitted model parameters would predict. We thus increased G by a factor of 2 in degus to allow entrainment to this LD cycle. To ensure masking effects were not altered by this change, we also decreased ν_{vb} by a factor of 2, since the retinal input to the VLPO is proportional to both G and ν_{vb}.

The values of these 8 parameters are provided in **Table S2**, along with human values determined in previous work for reference.

Activity Measure

The model provides a simple threshold definition of arousal state: wake for $Q_m > 1$ s^{-1} and sleep for $Q_m \leq 1$ s^{-1} [4]. In order to compare model output with behavioral measures such as feeding, activity, and body temperature, we developed a model proxy for activity. Since MA firing patterns are well correlated with vigilance and behavior [30,48], we defined activity as a function of Q_m. We first time-averaged Q_m using a sliding window of width $w = 10$ min. This was done for two reasons: (i) to provide an appropriate comparison with experimental measures of activity that are typically windowed on timescales of minutes or longer; and (ii) to account for time required for diffuse MA connections to modulate the behavioral state of the cortex. We then denoted windows in which $\langle Q_m \rangle_t$ exceeded some threshold, Q_m^{thr}, as bouts of activity. A value of $Q_m^{thr} = 2.0$ s^{-1} was selected to produce a simulated activity record visually similar to the body temperature record of Kas and Edgar [44]. However, we note that our general results are not reliant on this particular choice of Q_m^{thr}. **Figure S2** shows model simulations of **Figure 5B** performed with different values of Q_m^{thr}. In each case, all salient behaviors are preserved; the only difference is in the density of the activity bouts across the whole raster diagram, which is higher for lower values of Q_m^{thr}.

Simulations

In all simulations, we allowed 20 days to achieve entrainment (where applicable) and avoid transients. Specific details of the more complicated simulations of Kas and Edgar [44], and Edgar et al. [25] are given below.

Kas et al. (1999)

To simulate the Kas and Edgar [44] protocol, we used the degu parameter values in **Table S2**. The experiment depicted in **Figure 5A** was then replicated exactly, using 30 lux during light periods. The presence of a running wheel was simulated by inverting the signs of the parameters a and/or ν_{vb} to simulate circadian signal inversion and masking inversion, respectively. Activity bouts were plotted using the method described above, with $Q_m^{thr} = 2.0$ s^{-1}. Three model parameters were fitted specifically to the data. First, τ_c was set to 23.0 h to match the free-running period observed in DD. Second, we chose $\delta = 0.0$ to generate a similar daily activity duration under LD without a wheel as seen in the data. Third, we set $G = 74$ to achieve entrainment to LD.

Edgar et al. (1993)

We modeled the experiment of Edgar et al. [25] using the squirrel monkey parameter values shown in **Table S2**. To simulate SCN-lesioned animals, we used $a = 0$, which is equivalent to setting $F_s = 0$ in Eq. S7. The 500 lux LL protocol was replicated, allowing 20 days of initial entrainment to avoid transients, followed by 100 days of simulated data collection. The MA firing rate, Q_m, was used as a proxy

for sleep/wake and rest/activity patterns, with $Q_m > 1$ s^{-1} being defined as wake for raster plots. Three model parameters were fitted specifically to the data for the intact animal. First, τ_c was set to 25.0 h to match the observed free-running period under LL in the intact animal. Second, we chose $\delta = 2.9$ to match the amount of daily wakefulness observed in the intact animal. Third, we set $\chi = 22$ h to produce a primarily monophasic sleep pattern in the intact animal.

To compare the spectral components of the model's predicted activity pattern with that reported experimentally for drinking behavior, we computed the discrete Fourier transform of Q_m using Matlab's inbuilt fast Fourier transform function, *fft* (Matlab R2010b, Natick, MA). We then plotted the amplitudes of each spectral component as a function of period. For visual comparison, we normalized both data and simulations by the area under each curve for periods of 0-38 h, since this was the full range reported by Edgar et al. [25].

Edgar et al. estimated circadian time by analysis of sleep/wake data [25]. We followed their method, defining circadian time zero as activity onset (the first 30-min block with > 50% wakefulness). Additional data are required to determine whether the model also accurately reproduces phase angle differences between the sleep/wake cycle and other circadian markers, such as hormone secretion. Under SCN-lesioned conditions, we used the period calculated for intact animals to compute circadian time, since no measurable circadian rhythm existed.

References

1. Phillips AJK, Czeisler CA, Klerman EB (2011) Revisiting spontaneous internal desynchrony using a quantitative model of sleep physiology. J Biol Rhythms 26: 441-453.
2. Jewett ME, Kronauer RE (1998) Refinement of a limit cycle oscillator model of the effects of light on the human circadian pacemaker. J Theor Biology 192: 455-465.
3. St Hilaire MA, Klerman EB, Khalsa SBS, Wright Jr. KP, Czeisler CA, et al. (2007) Addition of a non-photic component to a light-based mathematical model of the human circadian pacemaker. J Theor Biol 247: 583-599.
4. Phillips AJK, Robinson PA (2007) A quantitative model of sleep-wake dynamics based on the physiology of the brainstem ascending arousal system. J Biol Rhythms 22: 167-179.
5. Phillips AJK, Robinson PA (2008) Sleep deprivation in a quantitative physiologically based model of the ascending arousal system. J Theor Biol 255: 413-423.
6. Phillips AJK, Robinson PA, Kedziora DJ, Abeysuriya RG (2010) Mammalian sleep dynamics: How diverse features arise from a common physiological framework. PLoS Comput Biol 6: e1000826.
7. Phillips AJK, Chen PY, Robinson PA (2010) Probing the mechanisms of chronotype using quantitative modeling. J Biol Rhythms 25: 217-227.

8. Crawford ML, Marc RE (1976) Light transmission of cat and monkey eyelids. Vision Res 16: 323-324.

9. Robinson J, Bayliss SC, Fielder AR (1991) Transmission of light across the adult and neonatal eyelid in vivo. Vision Res 31: 1837-1840.

10. Ando K, Kripke DF (1996) Light attenuation by the human eyelid. Biol Psychiatry 39: 22-25.

11. Kronauer RE, Forger DB, Jewett ME (1999) Quantifying human circadian pacemaker response to brief, extended, and repeated light stimuli over the photopic range. J Biol Rhythms 14: 500-515.

12. Kronauer RE (1990) A quantitative model for the effects of light on the amplitude and phase of the deep circadian pacemaker, based on human data. In: Horne J, editor. Sleep '90, Proceedings of the Tenth European Congress on Sleep Research. Dusseldorf: Pontenagel Press. pp. 306-309.

13. Refinetti R (2008) The diversity of temporal niches in mammals. Biol Rhythm Res 39: 173-192.

14. Cohen R, Kronfeld-Schor N, Ramanathan C, Baumgras A, Smale L (2010) The substructure of the suprachiasmatic nucleus: Similarities between nocturnal and diurnal spiny mice. Brain Behav Evol 75: 9-22.

15. Novak CM, Ehlen JC, Albers HE (2008) Photic and nonphotic inputs to the diurnal circadian clock. Biol Rhythm Res 39: 291-304.

16. Panda S, Hogenesch JB (2004) It's all in the timing: Many clocks, many outputs. J Biol Rhythms 19: 374-387.

17. Fleshner M, Booth V, Forger DB, Behn CGD (2011) Circadian regulation of sleep-wake behaviour in nocturnal rats requires multiple signals from suprachiasmatic nucleus. Philos Transact A Math Phys Eng Sci 369: 3855-3883.

18. Inouye ST, Kawamura H (1982) Characteristics of a circadian pacemaker in the suprachiasmatic nucleus. J Comp Physiol A 146: 153-160.

19. Sato T, Kawamura H (1984) Circadian rhythms in multiple unit activity inside and outside the suprachiasmatic nucleus in the diurnal chipmunk (*Eutamias sibiricus*). Neurosci Res 1: 45-52.

20. Kurumiya S, Kawamura H (1988) Circadian oscillation of the multiple unit activity in the guinea pig suprachiasmatic nucleus. J Comp Physiol A162: 301-308.

21. Nunez AA, Bult A, McElhinny TL, Smale L (1999) Daily rhythms of Fos expression in hypothalamic targets of the suprachiasmatic nucleus in diurnal and nocturnal rodents. J Biol Rhythms 14: 300-306.

22. Schwartz MD, Nunez AA, Smale L (2004) Differences in the suprachiasmatic nucleus and lower subparaventricular zone of diurnal and nocturnal rodents. Neuroscience 127: 13-23.

23. Robinson PA, Rennie CJ, Wright JJ (1997) Propagation and stability of waves of electrical activity in the cerebral cortex. Phys Rev E 56: 826-840.

24. Ibuka N, Kawamura H (1975) Loss of circadian rhythm in sleep-wakefulness cycle in the rat by suprachiasmatic nucleus lesions. Brain Res 96: 76-81.

25. Edgar DM, Dement WC, Fuller CA (1993) Effect of SCN lesions on sleep in squirrel monkeys: Evidence for opponent processes in sleep-wake regulation. J Neurosci 13(3): 1065-1079.

26. Easton A, Meerlo P, Bergmann B, Turek FW (2004) The suprachiasmatic nucleus regulates sleep timing and amount in mice. Sleep 27: 1307-1318.

27. Baker FC, Angara C, Szymusiak R, McGinty D (2005) Persistence of sleep-temperature coupling after suprachiasmatic nuclei lesions in rats. Am J Physiol Regul Integr Comp Physiol 289: R827-838.

28. Porkka-Heiskanen T, Strecker RE, McCarley RW (2000) Brain site-specificity of extracellular adenosine concentration changes during sleep deprivation and spontaneous sleep: an in vivo microdialysis study. Neuroscience 99: 507-517.

29. Imeri L, Opp MR (2009) How (and why) the immune system makes us sleep. Nat Rev Neurosci 10: 199-210.

30. Rajkowski J, Kubiak P, Aston-Jones G (1994) Locus coeruleus activity in monkey: Phasic and tonic changes are associated with altered vigilance. Brain Res Bull 35: 607-616.

31. Jewett ME, Forger DB, Kronauer RE (1999) Revised limit cycle oscillator model of human circadian pacemaker. J Biol Rhythms 14: 493-499.

32. Lu J, Zhang Y-H, Chou TC, Gaus SE, Elmquist JK, et al. (2001) Contrasting effects of ibotenate lesions of the paraventricular nucleus and subparaventricular zone on sleep-wake cycle and temperature regulation. J Neurosci 21: 4864-4874.

33. Chou TC, Scammell TE, Gooley JJ, Gaus SE, Saper CB, et al. (2003) Critical role of dorsomedial hypothalamic nucleus in a wide range of behavioral circadian rhythms. J Neurosci 23: 10691-10702.

34. Bailey TW, Nicol GD, Schild JH, DiMicco JA (2003) Synaptic and membrane properties of neurons in the dorsomedial hypothalamus. Brain Res 985: 150-162.

35. Zeitzer JM, Dijk DJ, Kronauer RE, Brown EN, Czeisler CA (2000) Sensitivity of the human circadian pacemaker to nocturnal light: Melatonin phase resetting and suppression. J Physiol (Lond) 526.3: 695-702.

36. Nelson DE, Takahashi JS (1991) Sensitivity and integration in a visual pathway for circadian entrainment in the hamster (*Mesocricetus auratus*). J Physiol 439: 115-145.

37. Agrawal DC, Leff HS, Menon VJ (1996) Efficiency and efficacy of incandescent lamps. Am J Phys 64: 649-654.

38. Trachsel L, Tobler I, Borbely AA (1986) Sleep regulation in rats: Effects of sleep deprivation, light, and circadian phase. Am J Physiol 251: R1037-1044.

39. Aston-Jones G, S. C, Zhu Y, Oshinsky ML (2001) A neural circuit for circadian regulation of arousal. Nat Neurosci 4: 732-738.

40. Saper CB, Scammell TE, Lu J (2005) Hypothalamic regulation of sleep and circadian rhythms. Nature 437: 1257-1263.

41. Adams PM, Barratt ES (1974) Nocturnal sleep in squirrel monkeys. Electroencephalogr Clin Neurophysiol Suppl 36: 201-204.

42. Gander PH, Moore-Ede MC (1983) Light-dark masking of circadian temperature and activity rhythms in squirrel monkeys. Am J Physiol 245: R927-R934.

43. Czeisler CA, Duffy JF, Shanahan TL, Brown EN, Mitchell JF, et al. (1999) Stability, precision, and near-24-hour period of the human circadian pacemaker. Science 284: 2177-2181.

44. Kas MJH, Edgar DM (1999) A non-photic stimulus inverts the diurnal-nocturnal phase preference in *Octodon degus*. J Neurosci 19: 328-333.

45. Aschoff J (1979) Circadian Rhythms: Influences of internal and external factors on the period measured in constant conditions. Zeitschrift fur Tierpsychologie 49: 225-249.

46. Muñoz-Delgado J, Fuentes-Pardo B, Baum AE, Lanzagorta N, Arenas-Rosas R, et al. (2005) Presence of a circadian rhythm in the spider monkey's (*Ateles geoffroyi*) motor activity. Biol Rhythm Res 36: 115-121.

47. Lu J, Shiromani PJ, Saper CB (1999) Retinal input to the sleep-active ventrolateral preoptic nucleus in the rat. Neuroscience 93: 209-214.

48. Aston-Jones G, Bloom FE (1981) Activity of norepinephrine-containing locus coeruleus neurons in behaving rats anticipates fluctuations in the sleep-waking cycle. J Neurosci 1: 876-886.
